# Pharmacologically increasing O-GlcNAcylation increases complexity of astrocytes in the dentate gyrus of TgF344-AD rats

**DOI:** 10.3389/fnagi.2025.1690410

**Published:** 2025-12-16

**Authors:** Melissa L. Garcia, Adam R. Denton, Nateka L. Jackson, Michael D. Scofield, Lori L. McMahon

**Affiliations:** 1 Department of Cell, Developmental and Integrative Biology, University of Alabama, Birmingham, AL, United States; 2 Department of Neuroscience, Medical University of South Carolina, Charleston, SC, United States; 3 Department of Psychology, Tusculum University, Tusculum, TN, United States

**Keywords:** astrocytes, microglia, hippocampus, O-GlcNAc, amyloid-beta, noradrenergic axons, Alzheimer’s disease, neurodegeneration

## Abstract

**Background:**

Alzheimer’s disease (AD) pathology begins two or three decades prior to the onset of cognitive symptoms and is characterized by amyloid-*β* (Aβ) and hyperphosphorylated tau (pTau) accumulation, reactive glial cells, increased inflammation, and neuronal degeneration in later stages. Preclinical studies report that increasing the post-translational modification, O-GlcNAcylation, involving the addition of a single N-acetylglucosamine (GlcNAc) moiety to serine or threonine residues, can reduce amyloidogenic processing of amyloid precursor protein (APP) and compete with serine phosphorylation on tau, decreasing hyperphosphorylated tau accumulation. Protein O-GlcNAcylation can have anti-inflammatory effects, suggesting the possibility that increasing O-GlcNAcylation may decrease reactive gliosis and other pathological changes in AD.

**Methods:**

This study aimed to assess the possible beneficial effects of pharmacologically enhancing O-GlcNAcylation by inhibiting O-GlcNAcase (OGA), the enzyme responsible for the removal of O-GlcNAc moieties, on progressive AD pathology using female TgF344-AD rats. The selective OGA inhibitor thiamet-G [TMG; 10 mg/kg, subcutaneously (s.c.)] was administered three times per week for 3 months starting at 6 months of age, a time point when Aβ pathology is evident in the hippocampus. Western blot analysis was used to measure protein levels of GFAP, Iba-1, and Aβ. Immunohistochemistry and confocal imaging were used to assess Aβ plaques, astrocyte and microglia complexity, and degeneration of tyrosine hydroxylase-positive (TH+) axons.

**Results:**

In TgF344-AD rats, we found significantly increased astrocyte complexity, defined as increased process length and branches, increased numbers of microglia, loss of noradrenergic axons (NA), and significant Aβ plaques compared to WT, confirming previous work by us and others. Notably, pharmacologically increasing O-GlcNAcylation further increased astrocyte complexity in TgF344-AD rats, specifically those located in close proximity to Aβ plaques, while microglia morphology and Aβ staining were unaffected. O-GlcNAcylation was not able to lessen the loss of TH + axons in TgF344-AD rats, although fewer dystrophic axons were observed, suggesting a possible beneficial effect.

**Discussion:**

Our findings demonstrate that increasing O-GlcNAcylation in TgF344-AD rats using a cyclical treatment protocol at a time when Aβ pathology is already significant does not provide broad beneficial effects on Aβ accumulation, microglial reactivity, or noradrenergic axon loss, although there appears to be fewer dystrophic axons. Importantly, increasing O-GlcNAcylation in TgF344-AD rats has dual beneficial effects on astrocyte reactivity. Astrocytes in close proximity to Aβ plaques are more complex with longer processes and more branches compared to those in saline-treated TgF344-AD rats at the same distance, enabling them to surround plaques and protect nearby neurons. Astrocytes located at more distal locations from plaques are less reactive than those at the same distance in saline-treated TgF344-AD rats, permitting a less pathological local environment for nearby neurons. Our findings offer new insights into the possible mechanisms that might contribute to the beneficial therapeutic effects of increasing O-GlcNAcylation during progressive AD pathology.

## 1 Introduction

Alzheimer’s disease (AD), a slowly progressing neurodegenerative disorder, is the most common cause of dementia globally (*Alzheimers Dement.*, 2024). Hallmark pathological changes begin 20–30 years prior to cognitive deficits and include the accumulation of amyloid-*β* (Aβ) and hyperphosphorylated Tau (pTau) in vulnerable brain regions such as the hippocampus, entorhinal cortex, and locus coeruleus (Andres-Benito et al., 2017; Braak and Del Tredici, 2012; Braak et al., 2011; Theofilas et al., 2018). This initiates a positive feedback loop that promotes neuroinflammation and gliosis, further accelerating disease progression, which ultimately leads to axon degeneration and neuronal loss (Theofilas et al., 2018; De Strooper and Karran, 2016; Doig, 2018; Faghihi et al., 2008; Webers et al., 2020). The difficulty in pinpointing the exact sequence of these pathological events makes it challenging to differentiate between causal pathogenic changes and compensatory responses.

Recent research has highlighted key roles for astrocytes and microglia in AD onset and progression (De Strooper and Karran, 2016; Brandebura et al., 2023; Chen et al., 2025; Liddelow and Barres, 2017; Liddelow et al., 2017; Liddelow et al., 2024; Taddei et al., 2023; Taddei et al., 2022). Under normal conditions, astrocytes exert essential homeostatic functions in modulating neuronal excitability, synaptic stability, transmission, and plasticity, as well as the integrity of the blood–brain barrier (Tan and Eroglu, 2021; Lalo et al., 2011; Gao et al., 2016; Martinez-Gallego and Rodriguez-Moreno, 2023; Lopez-Ortiz and Eyo, 2024; Fu et al., 2021). Microglia serve as the resident immune cells in the brain, protecting it from injury and infection, and they also modulate synaptic plasticity (Borst et al., 2021; Umpierre and Wu, 2021; Rodriguez-Gomez et al., 2020; Lochhead et al., 2020; Cornell et al., 2022). Astrocytes undergo complex morphological and functional changes in AD. Reactive astrocytes can surround Aβ plaques, where they provide a protective barrier against the toxic effects of Aβ (Sofroniew, 2015; Sofroniew, 2020; Rodriguez et al., 2009). They can also contribute to neuroinflammation and neuronal death due to loss of their homeostatic function and gain of toxic functions (Brandebura et al., 2023; Pekny and Pekna, 2014; Preman et al., 2021; Shah et al., 2022; Deng et al., 2024; Habib et al., 2020). Furthermore, recent evidence suggests that astrocytes in AD may act as antigen-presenting cells, further exacerbating the inflammatory responses (Rostami et al., 2020; Sutter and Crocker, 2022; Mitchener et al., 2025). Like astrocytes, microglia display dystrophic phenotypes and functions in AD models (Webers et al., 2020; Deng et al., 2024; Bachstetter et al., 2015; Wu et al., 2023; Sanchez-Mejias et al., 2016; Davies et al., 2017), and their crosstalk with astrocytes amplifies neuroinflammation (Chen et al., 2025; Lopez-Ortiz and Eyo, 2024; Deng et al., 2024; Matejuk and Ransohoff, 2020; Drago et al., 2017; Jo et al., 2017; Liu et al., 2024; Liu et al., 2011; Xie et al., 2020). The complex interaction between glial cells and their role in AD is still poorly understood, but their increased reactivity is clearly a hallmark of disease progression and a target for therapeutic intervention (Brandebura et al., 2023; Chen et al., 2025; Deng et al., 2024; Kim et al., 2024; Kraft et al., 2013; Leyns and Holtzman, 2017).

Another core feature of human AD is degeneration of locus coeruleus (LC) noradrenergic (NA) neurons and loss of NA innervation in terminal regions, particularly the hippocampus (Andres-Benito et al., 2017; Llorens et al., 2017; Kelly et al., 2017; Lin et al., 2024; Dahl et al., 2023). Booze et al. reported decreased frequency of long thin TH + axons in human AD brains but increased frequency of shorter, tortuous axons (Booze et al., 1993). This atypical morphology of NA axons has been reported previously in experimental rat models of cholinergic degeneration by others and confirmed by us (Scheiderer et al., 2006; Harrell et al., 2001; Nelson et al., 2014). The degree of NA degeneration is tightly associated with the transition from unimpaired to mildly cognitively impaired, to impaired (Dahl et al., 2023; Grudzien et al., 2007), and is correlated to early pTau accumulation in the LC (Dahl et al., 2022; Ohm et al., 2020). Importantly, loss of wake-promoting neurons in the LC could explain arousal deficiencies in AD patients as well as sundowning (Oh et al., 2019).

Identifying therapeutic approaches that can interrupt or otherwise limit the progression of AD pathology is critically needed. Gaining attention is the post-translational modification (PTM), O-GlcNAcylation, involving the addition of a single N-acetylglucosamine (GlcNAc) moiety to serine or threonine residues. O-GlcNAcylation is regulated by two enzymes: O-GlcNAc transferase (OGT), which adds GlcNAc, and O-GlcNAcase (OGA), which removes it (Chatham et al., 2021; Hart et al., 2007). These enzymes are highly abundant in regions associated with learning and memory, such as the hippocampus (Zhao et al., 2011). Pharmacologically or genetically increasing O-GlcNAcylation in both culture systems and transgenic rodent models can reduce amyloidogenic processing of APP (Jacobsen and Iverfeldt, 2011; Kim et al., 2013; Yuzwa et al., 2014), and compete with serine phosphorylation of tau, thereby preventing tau aggregation (Yuzwa et al., 2014; Bartolome-Nebreda et al., 2021; Graham et al., 2014; Hastings et al., 2017; Wang et al., 2011; Wang et al., 2020), and decrease astrocyte reactivity (Dong et al., 2023). In addition, in pTau mutant mice, increasing O-GlcNAc improves performance in hippocampus-dependent memory tasks (Yuzwa et al., 2014; Hastings et al., 2017; Wang et al., 2020). These preclinical findings have prompted clinical trials using OGA inhibitors for the treatment of AD (Bartolome-Nebreda et al., 2021; Selnick et al., 2019; Permanne et al., 2022). However, how pharmacologically increasing O-GlcNAcylation limits ongoing disease pathology remains to be elucidated, presenting a gap in current research that this study aims to address.

Here, using 6-month-old female Tg-F344AD rats, we assessed whether pharmacologically increasing O-GlcNAcylation using thiamet-G, an inhibitor of OGA, can limit astrocyte and microglia reactivity, lessen NA axon degeneration, and minimize Aβ accumulation in the dentate gyrus (DG) measured at 9 months of age, a time when significant Aβ pathology is present and when we have shown impaired synaptic function (Smith and McMahon, 2018; Goodman et al., 2021). In this study, we replicated previous findings of increased astrocyte and microglia reactivity using female TgF344-AD rats (Bac et al., 2023; Futacsi et al., 2025; Cohen et al., 2013; Rorabaugh et al., 2017). With the specific TMG dose and treatment schedule used, we found no significant effect on Aβ accumulation, microglia reactivity, or degeneration of NA axons, although fewer dystrophic axons were observed. Upon further analysis, we found that the effect of TMG on astrocyte morphology in rats was dependent upon their proximity to Aβ plaques, with those close to plaques being larger and more complex than in saline-treated TgF344-AD at the same distance to Aβ plaques, but becoming smaller and less complex at more distal sites compared to those in saline-treated TgF344-AD rats. These findings suggest dual beneficial effects of increasing O-GlcNAcylation on astrocytes, with those close to plaques having an enhanced ability to surround plaques and protect local neurons, while those at distal locations enable a healthier local environment.

## 2 Methods

### 2.1 Animals

All breeding and experimental procedures were approved by the University of Alabama at Birmingham (UAB) or the Medical University of South Carolina (MUSC) Institutional Animal Care and Use Committees, as experiments were conducted at both institutions, and followed the guidelines outlined by the National Institutes of Health. At UAB, rats were maintained under standard laboratory conditions [12 h reverse light/dark cycle, lights off at 6:00 h, 22 °C, 50% humidity, food (Harlan 2,916; Teklad Diets, Madison, WI), and water ad libitum]. Animals were housed using standard rat cages [7 in. (height) × 144 in2 (floor)]. At MUSC, rats were maintained under standard laboratory conditions [12 h reverse light/dark cycle, lights off at 6:00 h, 22 °C, 50% humidity, food (PicoLab Verified 75 IF LabDiet, and water ad libitum)]. Animals were housed in standard rat cages [~8 in. (height) x 140 in2 (floor)].

TgF344-AD males (originally obtained from Terrance Town at the University of Southern California), harboring the APP Swedish (APPswe) and delta exon 9 mutant human presenilin-1 (PS1ΔE9) transgenes, were bred to WT F344 females (Envigo). Transgene incorporation was verified by polymerase chain reaction (PCR) as described previously (Smith and McMahon, 2018). For experiments, female TgF344-AD and WT littermates were aged to 6 months of age, when AD pathology is already significant, but there are few cognitive changes.

### 2.2 Thiamet-G injections

#### 2.2.1 Dosing schedule

To determine the dosing schedule, control adult male Fisher 344 (F344) rats were purchased from Charles River at 8 weeks of age. Rats were injected subcutaneously via neck scruff between 12 and 20 weeks of age to allow time to recover before being injected with either 1 injection or 3 injections of thiamet-G (TMG; 10 mg/kg) over a week [Monday, Wednesday, Friday (MWF)] with rats euthanized and brains collected at 8 h, 24 h, 48 h, 72 h and 7 days after the last injection of TMG, with one saline-treated animal collected at each timepoint, for an *n* = 5 per group.

#### 2.2.2 WT and TgF344-AD rats treated with TMG between 6 to 9 months of age

**Figure 1:**
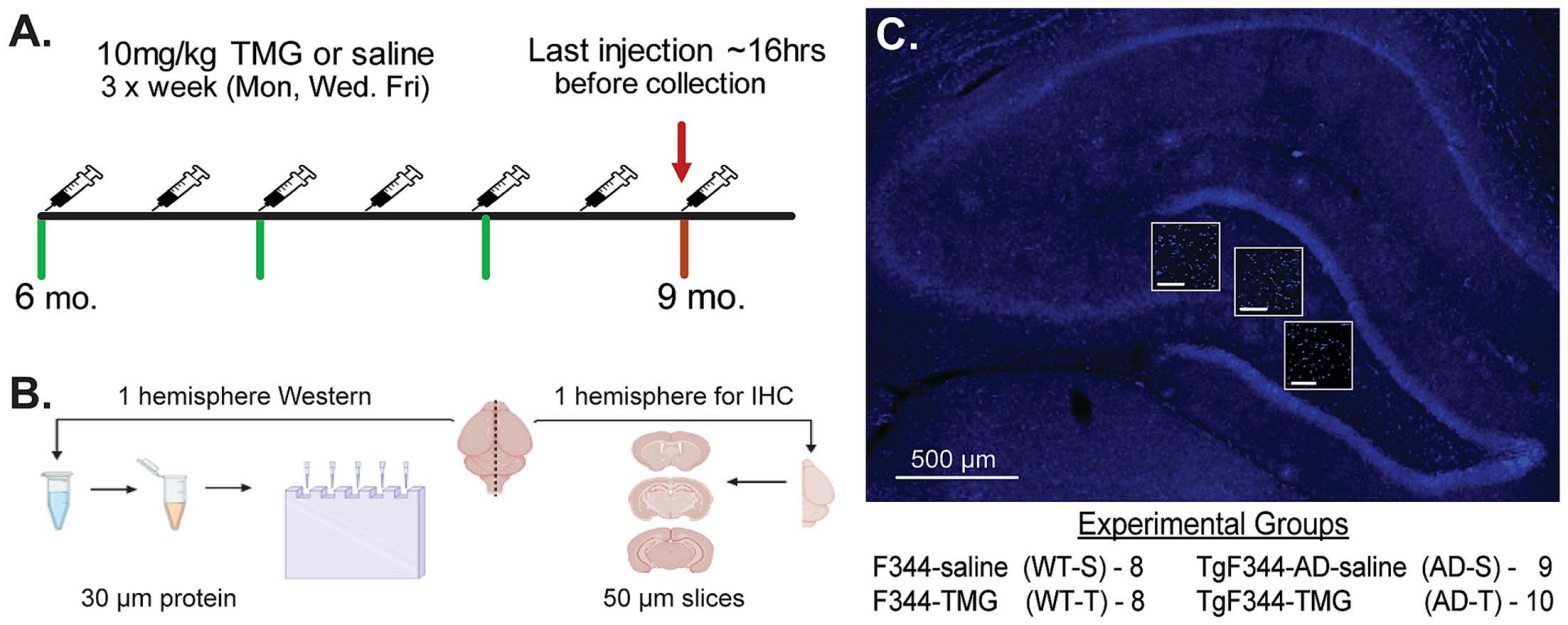
Thiamet-G (TMG) injection schedule and brain tissue processing. **(A)** Diagram illustrates the Monday, Wednesday, Friday (MWF) TMG (10 mg/kg) or saline injection schedule and tissue collection using female WT and TgF344-AD rats aged 6–9 months. Tissue was collected ~16 h after the last injection. **(B)** Schematic illustrating the use of hippocampus from one hemisphere with DG subdissected and flash frozen to be processed for Western blots using 30 μg protein. The other hemisphere was immediately dropped fixed in 4% PFA for 48 h before undergoing cryoprotection in 30% sucrose. Frozen sections were cut coronally into 50 microns (μm) slices using a freezing sliding microtome and stored in cryoprotectant until IHC processing. **(C)** Representative image illustrating the approximate regions in the hilus where confocal images were collected (63x) from each slice. Small scale bars = 100 μm. 4 experimental groups were used for this study: non-transgenic Fischer-344 rats injected with saline (WT-S, *n* = 8) or TMG (WT-T, *n* = 8) and transgenic Fischer-344-AD (TgF344-AD, AD) rats, injected with saline (AD-S, *n* = 9) or TMG (AD-T, *n* = 10). Source: https://BioRender.com/ghnetx7

Female Fisher transgenic-344-AD (TgF344-AD, AD) and non-transgenic (WT) littermates bred and aged in our colony were injected with TMG or saline subcutaneously via neck scruff on a MWF schedule from 6 to 9 months of age (Figure 1A). This age was chosen because TgF344-AD rats already have significant hippocampal Aβ pathology present at 6 months (e.g., Aβ plaques, reactive gliosis, degeneration of LC-NA axons; Goodman et al., 2021; Cohen et al., 2013; Rorabaugh et al., 2017). We tested the hypothesis that increasing O-GlcNAc after pathology is present can prevent or lessen further pathological changes. At the time of euthanasia, rats were anesthetized with isoflurane before undergoing cardiac perfusion with oxygenated artificial cerebrospinal fluid (ACSF; in mM as follows: 119.0 NaCl, 2.5 KCl, 1.3 MgSO4, 2.5 CaCl2, 1.0 NaH2PO4, 26.0 NaHCO3, 11.0 glucose). Following perfusion, rats were rapidly decapitated, and the brain was removed. Half of the brain was sub-dissected into regions of interest and flash frozen for Western blot analysis, while the other half was drop-fixed in 4% paraformaldehyde for 72 h (Figure 1B). Western blots were conducted using isolated DG, and confocal imaging was performed throughout the hilus (Figure 1C).

### 2.3 Western blot analysis

Rat hippocampi were sub-dissected into DG enriched samples, weighed while frozen, then homogenized in 20x their weight of TPER containing phosphatase and protease inhibitors (per product recommendation 1 tablet/10 mL, Pierce Phosphatase Inhibitor Mini Tablets cat# A32957), as well as TMG (1.24 mg/mL of TPER), using a BeadMill homogenizer (2.8 mm ceramic beads, speed = 4.85, time = 20 s, cycle x2, ensuring all tissue is completely homogenized). Samples were then centrifuged at 12,000 × g for 12 min before the lysates were collected. Lysate concentrations were determined via a BCA assay and diluted to 20-30 μg protein, depending on the protein of interest (20ug GFAP and Iba1, 30ug CTD110.6 and Aβ, Licor 4x loading buffer cat# 928–40,004). Western blots were performed using a Bio-Rad setup with hand-poured 7.5% (CTD 110.6) or 12% (GFAP, Iba-1, and Aβ) Bis-Tris gels. Each gel contained two ladders and a control group, which consisted of a blend of cerebellum from each animal to normalize the blots. Gels were run using a Bio-Rad tank with an initial voltage of 120 V for ~20 min, followed by 180 V on ice. Protein was transferred onto PVDF membranes (Millipore) using the Bio-Rad Turboblot transfer system, using the Bio-Rad Mixed MW setting. Total protein (TP) levels were measured using Li-Cor Total Protein (cat# 926–11,021) stain, according to the manufacturer’s instructions, for protein normalization. TP was imaged and removed before blocking and primary antibody incubation. Blots were blocked in Li-Cor Blocking buffer for 1 h after TP was removed. Primary antibody concentrations: CTD.110.6 (1:750, Cell Signaling cat# 9875S), Glial Fibrillary Acidic Protein (1:1000, GFAP-Abcam cat# ab53554), Iba-1 (1:500, Wako Fijifilm cat# 019–19,741), and Amyloid-*β* (1:1000, 1–16 Antibody, Biolegend cat # 803003); antibodies were diluted in Li-Cor antibody diluent (cat# 927–66,003) and incubated overnight at 4 °C on a rocker. Licor secondaries were used at the manufacturer’s recommendation of 1:10,000 at room temperature for 1 h on a rocker (Licor IRDye® cat#‘s 926–32214, 925–68,021, 925–32,280). Blots were imaged using a Li-Cor Odyssey M and quantified with Empiria Studios. All Western blots were performed in duplicates, and the average of the duplicates was used. All samples were normalized to total protein and then to the control sample to normalize across blots. CTD110.6 and total protein require the quantification of the entire lane of signal (all detectable signals), as both recognize multiple targets.

### 2.4 Immunohistochemistry

Following fixation and three 10-minute washes in 1x PBS that contains 2% trtionx-100 (2% PBST), brain hemispheres were sunk in 30% sucrose for cryoprotection before being flash frozen in isopentane on dry ice (−40 °C) and stored at −80 °C. Coronal serial sections (50 μm) were cut using a freezing sliding microtome and stored in antifreeze at −20 °C. For each antibody used, 2 coronal dorsal hippocampal slices were stained. Sections were washed 6 × 10 min in 2%PBST to remove all antifreeze, followed by blocking with a modified antibody signal enhancing recipe from Rosas-Arellano et al. (2% donkey serum, 50 mM glycine, 0.05% Tween20, 0.1% Triton X-100) and 0.1% BSA diluted in 2% PBST (Rosas-Arellano et al., 2016) for 1 h at room temperature on a shaker. Sections were then transferred to 1.5 mL Eppendorf tubes with 1 mL of antibody solution and rotated on a VWR multitube rotator at speed 7. Primary antibody concentrations: Glial Fibrillary Acidic Protein (1:1000 48-h incubation, GFAP-Abcam cat# ab278054), Iba-1 (1:500 48-h incubation, Wako Fijifilm), Tyrosine Hydroxylase (24-h incubation 1:1000, EMD Millipore, cat# ab152), and Amyloid-*β* (incubation time dependent on co-label, 1:400, 1–16 Antibody, Biolegend cat # 803003). It has been shown that TH fibers in the hippocampus colocalize with dopamine beta-hydroxylase-positive fibers (Moudy et al., 1993), and can serve as a homolog for NA fiber labeling, previously described (Goodman et al., 2021; Dyer-Reaves et al., 2019). After incubation, slices were washed 6 × 10 min in 2% PBST, followed by the addition of secondary antibody (abberrior star red nanobody cat# STRED-1010-50UG; GFAP, Iba1 and TH; Alexa Fluor 488 cat# A32766, Aβ) for 4 h on a shaker. Sections were then washed 5 × 10 min in PBST and a final 10-min wash in 1 × PBS before mounting with Everbright mounting media containing DAPI (Biotium cat# 23018).

### 2.5 Confocal imaging

One to two slices from each animal, with 3 regions within the hilus of the DG, were imaged and analyzed (Figure 1C). The results from each section were averaged into one value per animal. Z-stacks were acquired using a Leica SP8 laser scanning confocal microscope (Leica, Buffalo Grove, IL) equipped with argon (Ar 488 nm), krypton (Kr 568 nm), and helium-neon (He-Ne 633 nm) laser lines. Images were captured with a 10 × dry or 63 × oil immersion objective with a 1 × digital zoom factor. Acquisition settings included: 1024 × 1,024 frame size, 16-bit image resolution, a frame average of 2, and a 1-μm step size. Z-stacks typically ranged from 47 to 61 μm in depth, and image analyses were normalized to image depth where applicable.

### 2.6 Image analysis

Following confocal image acquisition, z-stacks were imported into Arivis Vision 4D software (Carl Zeiss Microscopy GmbH. (2025). arivis Cloud [Computer software]. www.apeer.com Version 2.2, image core 2.9.0.0, Arivis AG, Munich, Germany). For high-throughput analysis, semi-automated approaches were used to identify structures of interest; inaccurate or falsely recognized cells or axons were adjusted or removed by an investigator blind to experimental groups.

*Amyloid*-*β*: Aβ was co-labeled with either anti-GFAP, anti-Iba-1, or anti-TH. Z-stacks were imported into Arivis software, where an analysis pipeline denoised the images before an intensity threshold was applied; a size exclusion of 500 voxels was used to identify amyloid plaques. Total voxel count was normalized to image volume to account for slight variations in slice thickness between images. The average voxel count from all images for each animal was used to determine amyloid pathology for each animal (9–12 images per animal).

*Astrocytes*: To assess the morphology of astrocytes, 3 z-stacks of anti-GFAP-stained astrocytes (staining protocol above) from the inner hilus of the DG were imported into Arivis software and reconstructed into a 3-D model. Cell bodies were then manually placed, as GFAP does not stain the soma well and does not extensively colocalize with DAPI. Although GFAP does not stain the finer processes, we are using changes in GFAP+ volume, process length, and branching to infer a change in overall cell arborization, and increases in astrocyte volume inferred from GFAP staining as increased reactivity, with increases in process length and branching as indicators of increased complexity. A semi-automated approach was then used to reconstruct cells using the Probabilistic reconstruction algorithm in NeuronTracer. Tubularity sensitivity and local threshold were determined from the image, and the seed filter set to 75%. The total number of cells within a field, as well as cell volume, total process length, and branch point number were exported. For each animal, we took the average of all astrocytes from 3 images to produce average cell values (total astrocytes analyzed: 4786: WT-S: 1284, WT-T: 1123, AD-S: 1083, AD-T: 1296). To assess if differences in astrocyte morphology characteristics were related to distance from Aβ plaques, we first measured both independently. Next, the distance from each astrocyte to the nearest Aβ plaque was measured in Arivis software and plotted in GraphPad.

*Microglia*: To assess the morphology of microglia, 3 z-stacks of anti-Iba-1-stained microglia (staining protocol above) from the inner hilus of the DG were imported into Arivis software and reconstructed into a 3-D model. A software pipeline was then created to identify DAPI and Iba-1 co-localization to accurately detect the cell body of microglia. A semi-automated approach was then used to reconstruct cells using the Probabilistic reconstruction algorithm in NeuronTracer. Tubularity sensitivity and local threshold were determined from the image, and seed filter set to 70%. The total number of cells within a field, as well as cell volume, total process length, and branch point number were exported. For each animal, we took the average of all microglia from 3 images to produce average cell values (total microglia analyzed: 1686, WT-S: 252, WT-T: 267, AD-S: 576, AD-T: 591). To assess if differences in microglia morphology characteristics were related to distance from Aβ plaques, we first measured both independently. Next, the distance from each microglia to the nearest Aβ plaque was measured in Arivis software and plotted in GraphPad.

*TH+ axons*: To assess TH + axons, 3 z-stack images from the inner hilus of the DG from 2 coronal slices were imported into Arivis software, and reconstructed into a 3-D model before an auto-threshold of 50 was applied as the lower intensity range minimum. This was adjusted, if necessary, by a blinded experimenter as necessary to correct erroneous masking. Total voxel count was measured and normalized to the total image volume to account for the total innervation. During imaging and quantification, the observation was made that some images contained large, thickened, and dystrophic (blebbed) axons, commonly referred to as torturous axons (Booze et al., 1993). In an attempt to quantify the blebbed axons, a size exclusion of 250 voxels was used to identify these abnormalities; all flagged objects over 250 voxels were examined to ensure only torturous axons were included in quantification.

### 2.7 Statistical analysis

Unless otherwise noted, standard Analysis of Variance (ANOVA) was used for all statistical analyses. Values of technical replicates (i.e., individual cells) were averaged together to create animal averages to avoid violation of the Assumption of Independence and to thus avoid Type I error inflation (Festing and Altman, 2002). Animal genotype and treatment conditions were evaluated as between-subject factors across all analyses, with both main effects and interactions examined. To interrogate our hypothesis that pharmacologically increasing O-GlcNAcylation via TMG could limit astrocyte/microglia reactivity, simple effects tests were employed to examine treatment group differences between genotype and treatment conditions (i.e., WT Saline vs. AD TMG). Bonferroni corrections were applied to all simple effects tests to control for family-wise Type I error rates (Nicholson et al., 2022). To evaluate astrocyte/microglia morphology with respect to Aβ plaque distance, separate linear regression models were fit for each condition (AD Saline vs. AD TMG). Parameters of constructed models (i.e., slope, y-intercept) were then tested to see if constructed models differed significantly between treatment groups (Denton et al., 2021; Denton et al., 2019). Animal weight data were analyzed with a mixed model ANOVA to investigate the relationship between rodent genotype, sex, and weight each week. For evaluation of Aβ immunohistochemistry, a standard t-test was employed as WT animals were not examined in this analysis. A *p*-value of *p* < 0.05 was considered the cutoff for statistical significance. All analyses were performed in IBM SPSS (v30) or GraphPad Prism (v11). All figures were made in GraphPad Prism (v11).

## 3 Results

### 3.1 Optimization of thiamet-G dosing schedule to increase O-GlcNAcylation

**Figure 2:**
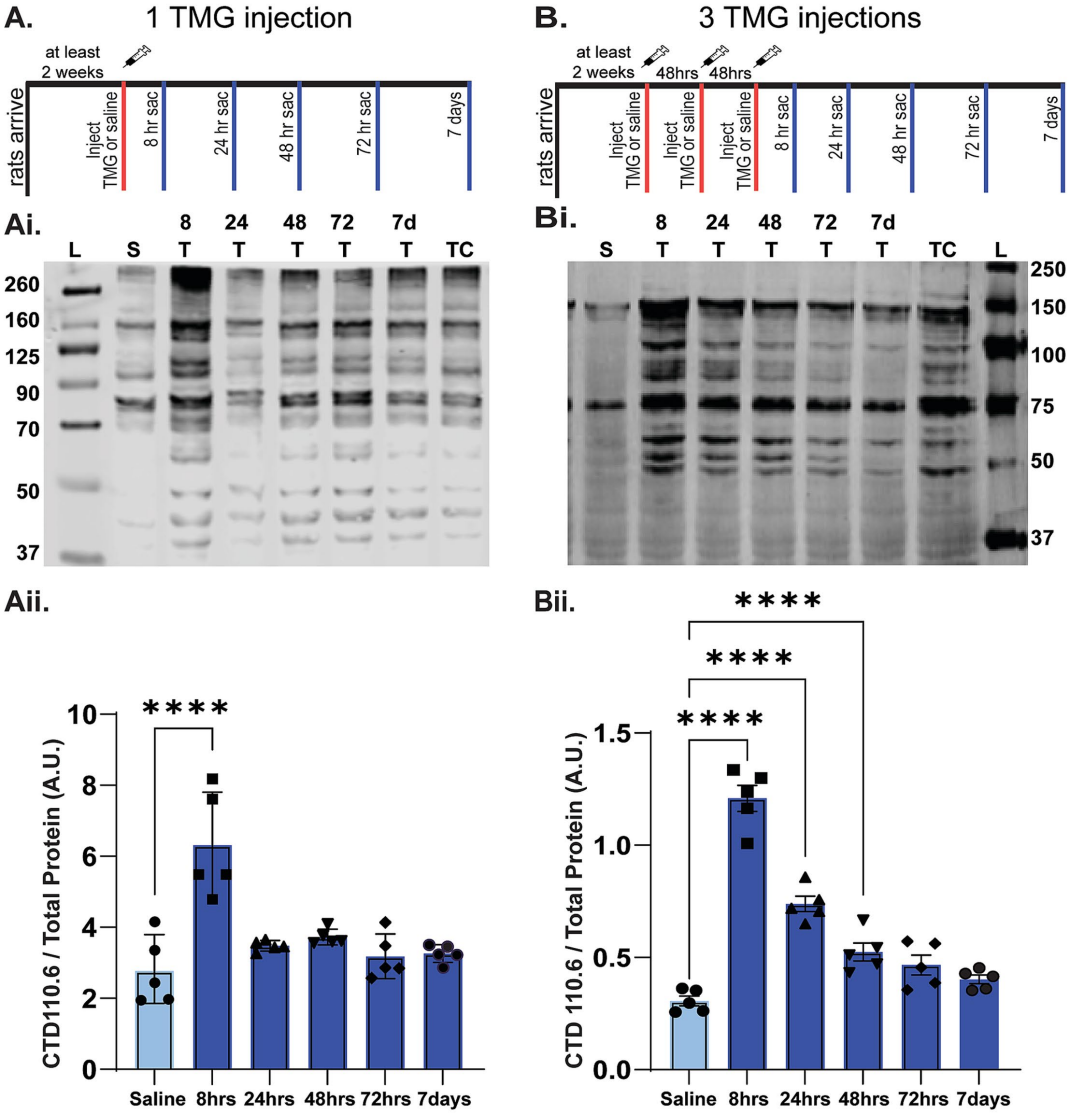
Time course of the increase in O-GlcNAcylation in whole hippocampal homogenate following in vivo TMG injection. **(A)** Diagram showing TMG injections (subcutaneous, s.c.) and hippocampal tissue collection using adult male Fisher rats injected with either one **(A)** or three **(B)** TMG (10 mg/kg) or saline injections. Red tick mark(s) denote when injections occurred. Blue tick marks denote when rats were euthanized post-injection: 8, 24, 48, 72 h or 7 days. (**Ai,Bi**; See Supplementary Figure 2 for full blots and TP). Representative Western blot (30 μg protein/lane) of O-GlcNAcylated proteins detected using the pan-O-GlcNAc antibody, CTD 110.6. Blot labeling left to right: Ladder (L), saline (S), 8 T, 24 T, 48 T, 72 T, and 7d indicate time of euthanasia in hrs or days (d) post-TMG injection. TC denotes cerebellum tissue used to normalize across blots for quantification; all lanes were normalized to this value after total protein. **(Aii,Bii)** Quantification of Western blots (*n* = 5 per group, 1 saline animal collected at each TMG timepoint, AU indicates arbitrary units). **(Aii)** Following a single TMG injection O-GlcNAcylated proteins are significantly increased at 8 h post-injection compared to saline-injected control rats. RMANOVA: F (5-24)=13.334, *p* < 0.01; pairwise comparisons: *p* < 0.001 at 8 h post-injection; *p* = 1.00 at all other post-injection times. **(Bii)** Results show 3 injections in a week significantly increase O-GlcNAcylation for up to 72 h after the last injection. RMANOVA: F (5,24)=71.967, *p* < 0.001; Pairwise comparisons: *p* < 0.001 at 8 h; *p* < 0.001 at 24 h; *p* = 0.008 at 48 h; *p* = 0.109 at 72 h; *p* > 1.00 at 7 days.

Before testing whether increasing O-GlcNAcylation can slow progression of AD pathology in TgF344-AD rats, our initial goal was to determine an optimal thiamet-G (TMG) dosing schedule that would allow O-GlcNAc levels to cycle. Because this PTM plays a vital role in many mechanisms involved in cell homeostasis (Chatham et al., 2021; Hart et al., 2011), prolonged, chronic increases in O-GlcNAcylation can have detrimental effects (Chatham et al., 2021; Banerjee et al., 2016; Karunakaran and Jeoung, 2010; Umapathi et al., 2021). We first determined the time course of the increase in O-GlcNAcylation following a single dose of TMG (10 mg/kg s.c.) compared to saline control using adult male Fisher 344 rats (*n* = 5/group). We euthanized the rats at 8 h, 24 h, 48 h, 72 h, and 7 days post-injection and assessed O-GlcNAc levels by Western blot using the pan-O-GlcNAc antibody, CTD 110.6, as we have done previously (Stewart et al., 2020; Taylor et al., 2014). As shown in Figure 2A, O-GlcNAc levels were significantly increased at 8 h post-injection and returned to baseline by 24 h (*p* < 0.001 at 8 h post-injection; *p* > 1.00 at all other post-injection times).

With the return of O-GlcNAc levels by 24 h following a single injection, we next sought to determine if there was a cumulative effect of administering TMG 3 times at a 48 h interval on Monday, Wednesday, and Friday (MWF). Therefore, we injected male rats on MWF and euthanizeed at 8 h, 24 h, 48 h, 72 h, and 7 days following the 3^rd^ injection. As shown in Figure 2B, O-GlcNAc levels peaked at 8 h, and precipitately decreased until it was no longer significantly elevated at 72 h post the 3^rd^ injection (RMANOVA; *p* < 0.001 at 8 h; *p* < 0.001 at 24 h; *p* = 0.008 at 48 h; *p* = 0.109 at 72 h; *p* = 1.00 at 7 days). Based upon these results, we determined that the optimal injection schedule for maximizing O-GlcNAc cycling without causing significant saturation or a complete return to baseline was a MWF injection schedule, which was used throughout the study.

### 3.2 Thiamet-G elicits similar increases in O-GlcNAcylation in WT and TgF344-AD rats

**Figure 3:**
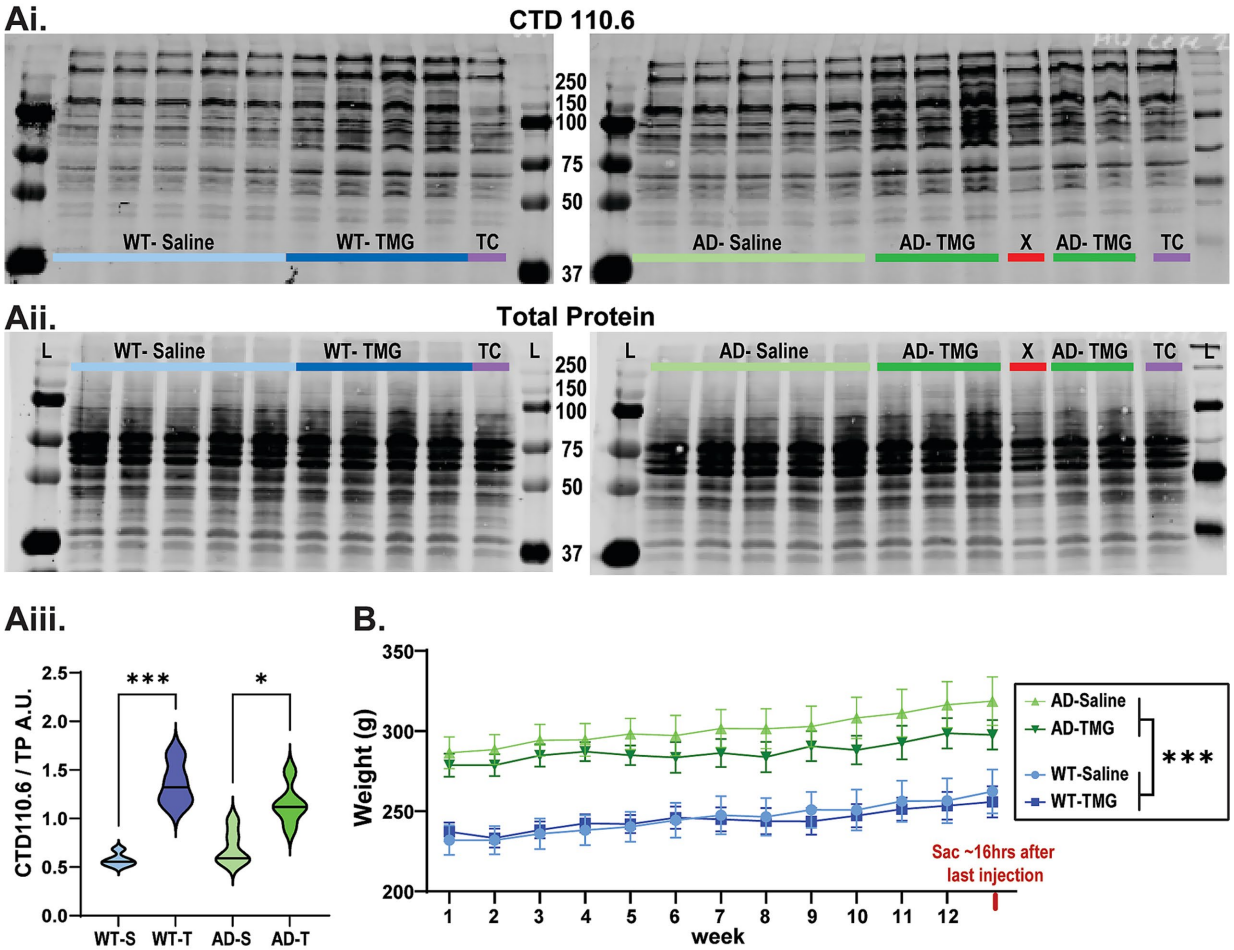
O-GlcNAc is significantly increased following a three-month TMG treatment protocol with no effect on body weight. **(Ai,Aii)** Representative Western blots displaying O-GlcNAcylated proteins detected using CTD 110.6 **(Ai)** and total protein TP for normalization **(Aii)** from WT (left) or TgF344-AD (right) rats injected with saline or TMG 3x per week for 3 months between 6 and 9 months of age. Rats are euthanized approximately 16 h post final injection. Experimental groups noted as: WT- saline (WT-S), WT-TMG (WT-T), TgF344-AD saline (AD-S), and TgF344-AD-TMG (AD-T) in this and all subsequent figures. (**Ai**, left) Representative blots of O-GlcNAcylated proteins from WT (**Ai,Aii**, left blots) and TgF344-AD rats (**Ai,Aii**, right blots): (X) is a sample prepared with no TMG in the lysate, (TC) denotes cerebellum control tissue used to normalize across blots for quantification; all lanes were normalized to this value after TP. **(Aiii)** Plot shows a significant increase in O-GlcNAcylation in TMG-treated rats [Effect of treatment: F (1,15)=55.215, *p* < 0.001] with no difference between genotypes [Effect of genotype: F (1,15)=0.229, *p* < 0.639], *n* = 2–4 rats per group. **(B)** Weight tracking data showing TgF344-AD rats are significantly heavier than WT rats before injections begin and persist throughout the treatment period [Genotype effect: F (1,31)=35.489, *p* < 0.001]. TMG did not significantly affect the weight of rats of either genotype: F (1,31)=0.499, *p* = 0.485. An interaction between genotype and treatment was also not found: F (1,31)=0.748, *p* = 0.394.

Next, using the MWF dosing schedule, we set out to test the hypothesis that pharmacologically increasing O-GlcNAc would slow progressive AD pathology in the DG of TgF344-AD rats. TMG (10 mg/kg, sc) or saline was delivered MWF each week for 3 months to female WT and Tg-F344-AD rats between 6 and 9 months of age (Figure 3). We specifically focused on the DG since our previous work showed the earliest alterations in synaptic function occur at excitatory synapses at medial-perforant path-dentate granule cell synapses (MPP-DGC) prior to CA1 (Smith and McMahon, 2018). The DG in one hemisphere was used for Western blot analysis, and the other for immunohistochemistry (IHC) to investigate possible beneficial effects on slowing disease progression. Rats were euthanized ~16 h following the last injection, and elevated O-GlcNAc levels were confirmed by Western blot (Figure 3A). We found no significant differences in basal O-GlcNAc levels between WT and TgF344-AD rats at 9 months (*p* = 0.275). In both WT and TgF344-AD rats, TMG induced significant increases in O-GlcNAcylation (WT *p* < 0.001; TgF344-AD *p* = 0.001), and the magnitude was not different between genotypes (*p* = 0.105). We did note that across all weeks, TgF344-AD rats weighed significantly more than their WT counterparts (*p* < 0.001), and there was no significant effect of increasing O-GlcNAc on body weight (*p* = 0.485; Figure 3C).

### 3.3 Pharmacologically increasing O-GlcNAcylation in TgF344-AD rats does not affect aβ accumulation

**Figure 4:**
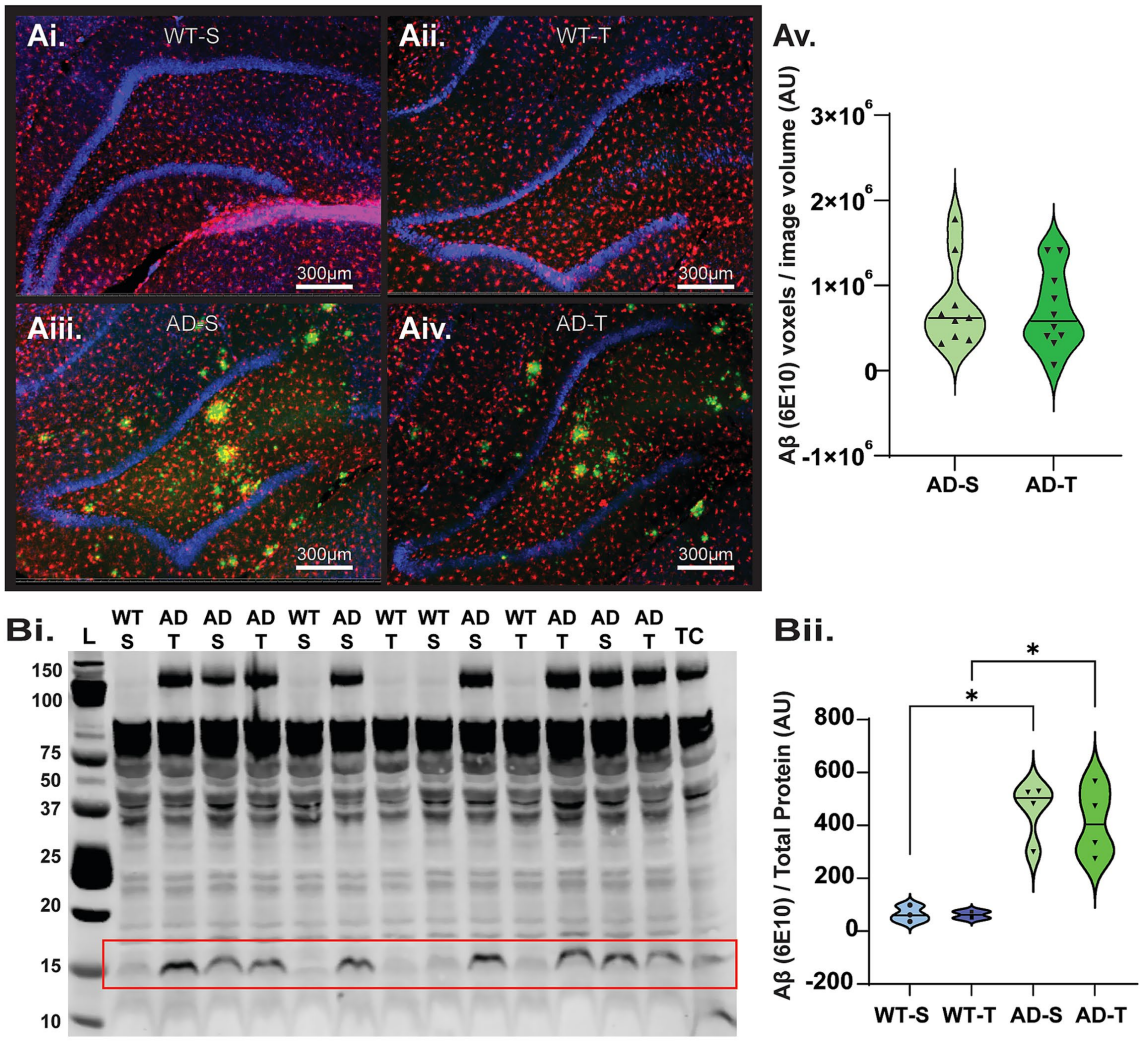
Increasing O-GlcNAcylation has no effect on Aβ accumulation in the DG. **(Ai–Aiv)** Representative confocal images of Aβ plaques detected (1–16; Biolegend, green) in the DG. In these images, microglia were also stained using anti-Iba-1 antibody (Wako/ Fujifilm, red), and the DNA stain DAPI (blue), scale bars = 500 μm. **(Av)** Quantification of average Aβ staining from all images (*N* = 8–10 rats per group, *n* = 7–8 pictures per rat), shown in total amyloid voxel volume in saline vs. TMG-treated rats [Treatment effect: F (1,17)=1.178, *p* = 0.819]. **(Bi)** Representative Western blot showing 15 kDa isoform of Aβ, denoted by the red box, TC denotes cerebellum technical control, (See Supplementary Figure 3 for full blots and TP, AU indicates arbitrary units). **(Bii)** Plot shows TgF344-AD rats have a significant increase in 15 kDa Aβ compared to WT, regardless of treatment [Genotype effect: F (1,9)=41.808, *p* < 0.001 *n* = 2–4 per group], no effect of treatment F (1,9)=0.187, *p* = 0.676, and no overall interaction effect F (1,9)=0.149, *p* = 0.708.

Aβ plaque accumulation is a key hallmark of progressive AD pathology. Some reports have shown that pharmacologically increasing O-GlcNAc through OGA inhibition decreases Aβ accumulation in cell models and in transgenic AD mice (Jacobsen and Iverfeldt, 2011; Kim et al., 2013; Griffith et al., 1995). Therefore, using 6-month-old female TgF344-AD rats, we first asked whether Aβ plaque load is decreased following a 3-month TMG treatment until rats reached 9 months of age. Using IHC (Figures 4Ai–Av), we observed no effect of TMG treatment on Aβ plaque voxel count when comparing to saline-treated TgF344-AD rats (Figure 4Av; *p* > 0.8) or in Western blot analysis probing the 15 kDa isoform (Figures 4Bi, ii; *p* > 0.5). Thus, any improvement found in other measures with TMG treatment will occur independently of a decrease in Aβ plaque load. Our results indicate that the dose (10 mg/kg), treatment schedule (3 times/week), and duration of TMG treatment (3 months) are ineffective in decreasing Aβ accumulation in female TgF344-AD rats once the pathology has already commenced.

### 3.4 Western blot does not reveal an effect of genotype or treatment on GFAP expression, while Iba-1 expression is increased in TMG-treated TgF344-AD rats

Previous studies have reported that hippocampal astrocytes and microglia in TgF344-AD rats are reactive as early as 6 months of age (Cohen et al., 2013; Rorabaugh et al., 2017; Chaney et al., 2021). Recent studies show acutely increasing O-GlcNAcylation decreases GFAP mRNA in control mice (Bell et al., 2025), suggesting decreased astrocyte reactivity even in healthy conditions. In transgenic 5x FAD mice, pharmacologic or genetic OGA inhibition decreases GFAP expression and reactive astrocytes and improves memory (Kim et al., 2013; Park et al., 2021). Also, in 5xFAD mice, OGA inhibition recovered phagocytic activity of microglia (Park et al., 2021). In *ogt* deficient mice, where O-GlcNAcylation is absent, GFAP expression is increased and astrocytes are reactive (Dong et al., 2023). Collectively, these data show that O-GlcNAc modulates glial cell reactivity such that increasing O-GlcNAc decreases astrocyte reactivity in both control and AD models.

**Figure 5:**
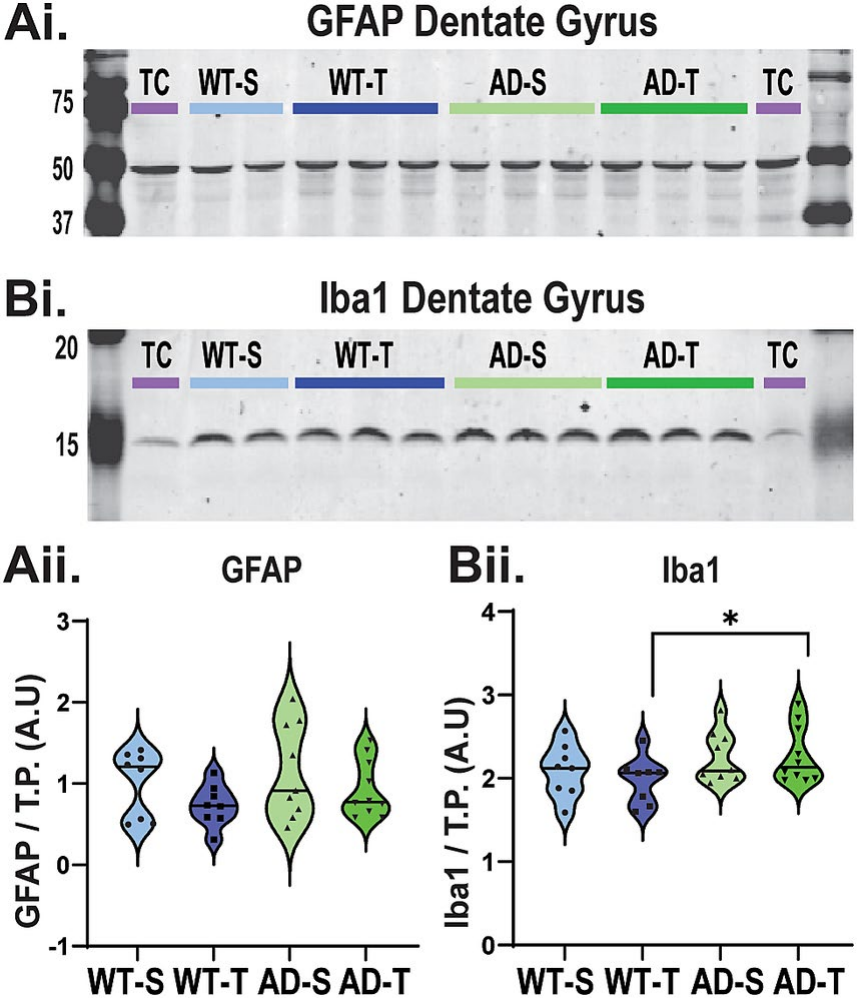
Western blot of DG does not reveal an effect of genotype or treatment on GFAP expression, while Iba-1 expression is increased in TMG-treated TgF344-AD rats. **(Ai)** Representative Western blot detecting GFAP (~50 kDa) protein in the DG (30 μg protein) from WT and TgF344-AD rats (See Supplementary Figure 4 for full blots and TP). **(Aii)** All lanes were normalized to their respective TP, and then blots were normalized using a cerebellum technical control (purple TC). AU indicates arbitrary units. No significant genotype or treatment effect on total GFAP protein in the DG [genotype effect: F (1,31)=1.433, *p* = 0.24; treatment effect: F (1,31)=2.913, *p* = 0.098; interaction effect: F (1,31)=0.56, *p* = 0.814]. **(Bi)** Representative Western blot detecting Iba-1(~15 kDa) protein in the DG (30 μg). **(Bii)** Significant genotype effect on Iba-1 protein in the DG [F (1,31)=4.872, *p* = 0.035]. While the overall interaction effect between genotype and treatment was not significant: F (1,31)=0.432, *p* = 0.516, Bonferroni-corrected pairwise comparisons show a significant difference between WT and AD saline-treated animals (*p* = 0.049); no main effect of treatment was observed [F (1,31)=0.165, *p* = 0.687].

To test for a possible benefit of increasing O-GlcNAcylation on glial cell reactivity, we first used Western blot to determine if expression of GFAP, an astrocyte marker, and expression of Iba-1, a microglia marker, are increased in the DG of TgF344-AD rats, and if these levels are decreased by TMG treatment. As shown in Figure 5A, expression of GFAP is not increased in TgF344-AD rats compared to WT (*p* = 0.24), and we find no overall effect of treatment (*p* = 0.098). However, regarding Iba-1 expression (Figure 5B), we do find a significant effect of genotype (Figure 5Bii, *p* = 0.035), with a simple effects test revealing an increase in Iba-1 expression in TMG-treated TgF344-AD rats compared to TMG-treated WT rats (*p* = 0.049). These results indicate that expression of GFAP and Iba-1 are differentially regulated in TgF344-AD rats, and they are differentially modulated by O-GlcNAcylation.

### 3.5 Increasing O-GlcNAcylation increases astrocyte complexity in TgF344-AD rats

**Figure 6:**
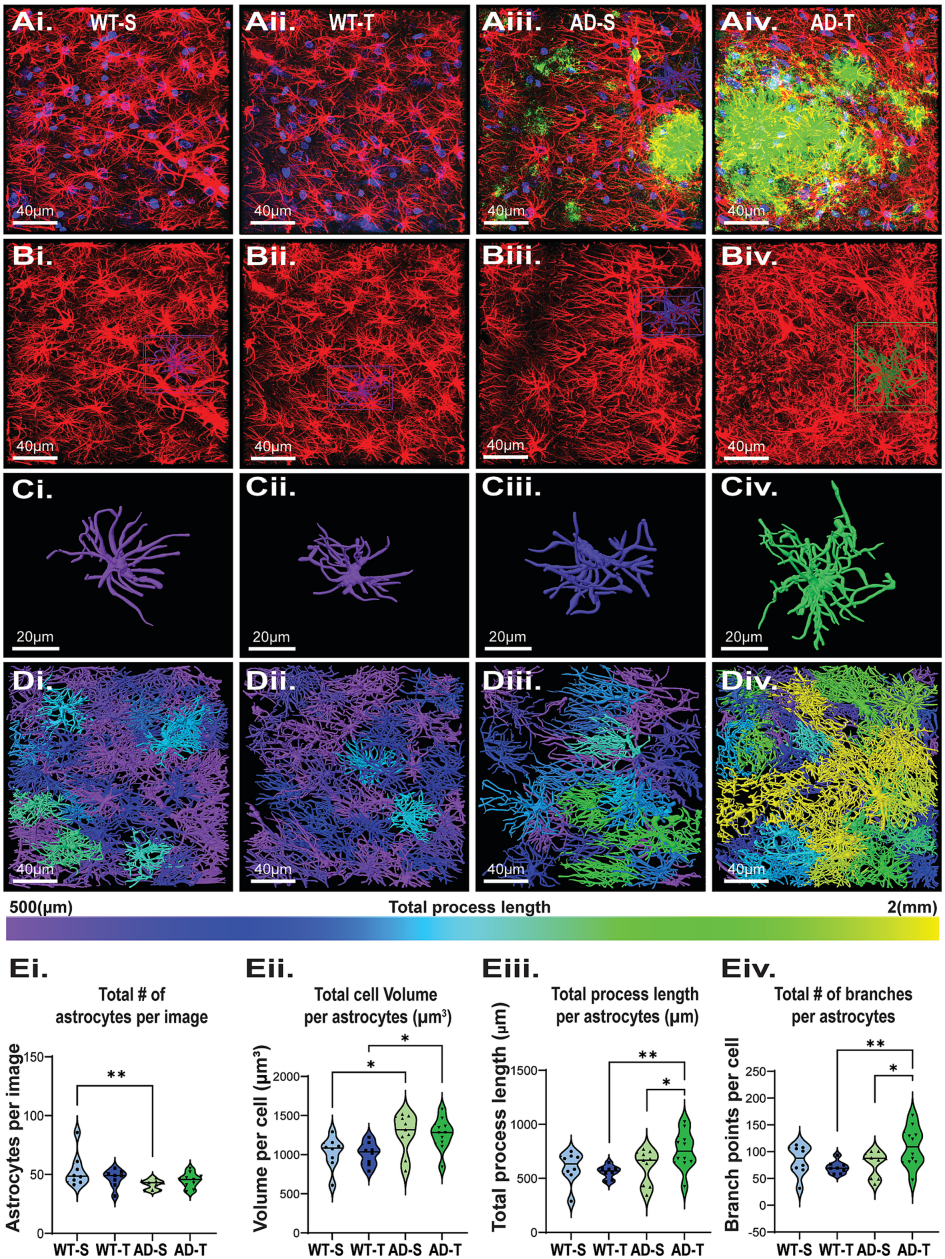
Increasing O-GlcNAc increases astrocyte process length and branching in TgF344-AD rats. **(Ai–Div)** Representative confocal images of astrocytes in the DG from all 4 experimental groups with morphological reconstruction using Arivis (Carl Zeiss Microscopy GmbH. (2023). arivis Cloud). **(Ai–Aiv)** Dapi (blue), GFAP (red), Aβ (green), scale bar = 40 μm. **(Bi–Biv)** Images of isolated GFAP staining; scale bar = 40 μm. **(Ci–Civ)** Examples of individual reconstructed astrocytes. Image color based on total process length of that cell, scale bar = 20 μm. **(Di–Div)** Full image Arivis reconstruction; cell color based on total process length shows an increase in the number of cells with longer total process lengths (green and yellow) in TgF344-AD rats. **(Ei–Eiv)** Graphs quantifying morphology attributes of astrocytes in the DG. Each dot represents 1 rat with an average of 3 images per animal. Total astrocytes analyzed: 4786, with a breakdown of WT-S: 1284, WT-T: 1123, AD-S: 1083, AD-T: 1296, *N* = 8–10 animals per group. **(Ei)** Significant effect of genotype on total number of GFAP positive cells with less in TgF344-AD rats, and no effect of treatment [significant genotype effect: F (1,31)=5.561, *p* = 0.025; no treatment effect: F (1,31)=0.165, *p* = 0.687; pairwise comparisons show a significant difference between saline-treated WT and AD rats (*p* = 0.008)]. **(Eii)** Significant effect of genotype on total astrocyte volume with increased volume in TgF344-AD rats [significant genotype effect: F (1,31)=12.364, *p* = 0.001; no effect of treatment: F (1,31)=0.009, *p* = 0.926; pairwise comparisons show a significant difference between saline-treated WT and AD rats (*p* = 0.023) and TMG-treated WT and AD rats (*p* = 0.015)]. **(Eiii)** Significant effect of genotype on process length with longer length in TgF344-AD rats [significant genotype effect: F (1,31)=4.182, *p* = 0.049; No treatment effect: F (1,31)=1.341 *p* = 0.256. A near interaction of treatment and genotype: F (1,31)=4.033, *p* = 0.053]. Pairwise comparisons show a significant difference between TMG-treated WT and AD rats (*p* = 0.007), as well as a significant increase between TMG-treated and saline-treated TgF344-AD rats (*p* = 0.026). **(Eiv)** While we observed no effect of genotype or treatment alone we did see a significant genotype x treatment interaction for number of branches, with TMG-treated TgF344-AD rats having more branches than TMG-treated WT rats [Genotype effect: F (1,31)=2.857, *p* = 0.101; Treatment effect: F (1,31)=1.124, *p* = 0.297; Genotype X treatment effect: F (1,31)=6.079, *p* = 0.019]; pairwise comparisons reveal a significant difference between TMG-treated WT and TgF344-AD rats, with AD-T cells having significantly more branches than WT-T (*p* = 0.006) as well as a significant increase in the number of branches in AD-T compared to AD-S (*p* = 0.014).

Astrocyte morphology can be altered even when GFAP expression is unchanged (Baldwin et al., 2024). Furthermore, astrocyte morphology is a more sensitive measure to infer function than bulk DG homogenate GFAP expression levels, as this allows measurements of individual cell changes (Sofroniew, 2020; Lana et al., 2019; Lee et al., 2022; Zhou et al., 2019; Sofroniew, 2014). Therefore, we used IHC and confocal imaging to assess changes in astrocyte morphology between WT and TgF344-AD rats and the possible benefit of increasing O-GlcNAcylation on reducing reactivity (Figure 6).

Brief inspection of the IHC data shows that astrocytes are more reactive in TgF344-AD rats compared to WT (Figures 6Ai–Div), consistent with published literature (Cohen et al., 2013; Rorabaugh et al., 2017; Chaney et al., 2021). Statistical analysis reveals significantly fewer astrocytes in the hilus of the DG in TgF344-AD rats compared to WT (*p* = 0.025; Figure 6Ei) with no effect of treatment (*p* = 0.587). Importantly, we found increased astrocyte volume in TgF344-AD rats compared to WT (*p* < 0.001) in both the saline (*p* = 0.023) and TMG-treated groups (*p* = 0.015; Figure 6Eii), with no genotype versus treatment interaction (*p* = 0.926). This finding is consistent with increased astrocyte reactivity, and the increased volume offsets the decrease in total number of astrocytes in TgF344-AD rats (Figures 6Ei,Eii). We next evaluated process length as an indicator of increased reactivity. We found a significant effect of genotype (*p* = 0.049; Figure 6Eiii) and a nearly significant interaction with TMG treatment (*p* = 0.053). TMG-treated TgF344-AD rats had significantly longer total process length compared to TMG-treated WT rats (*p* = 0.007). Also important is the finding that within the TgF344-AD group, astrocytes in TMG-treated rats had significantly longer total process length compared to saline-treated TgF344-AD rats (*p* = 0.026; Figure 6Eiii). Finally, while not finding a significant genotype effect in total branch point number (*p* = 0.101), we found a significant interaction between genotype and treatment (*p* = 0.019; Figure 6Eiv), with a significant difference between WT and TgF344-AD TMG-treated groups (*p* = 0.006) and between saline- and TMG-treated TgF344-AD groups (*p* = 0.014). Thus, unexpectedly, we found that TMG treatment significantly increases both process length and branch points only in TgF344-AD rats, with astrocytes in WT rats being resistant to these morphological changes. The longer process length and increased branch points in TMG-treated TgF344-AD rats indicate that increasing O-GlcNAcylation makes astrocytes more reactive. Furthermore, our results indicate that while there is an overall loss of astrocytes in TgF344-AD rats, they are compensating for this loss by increasing cell volume and complexity, and that increasing O-GlcNAc is not preventing or reversing astrocyte reactivity as hypothesized, but rather it is exacerbating reactivity.

### 3.6 Astrocytes in thiamet-G-treated TgF344-AD rats located proximal to aβ plaques are more reactive

**Figure 7:**
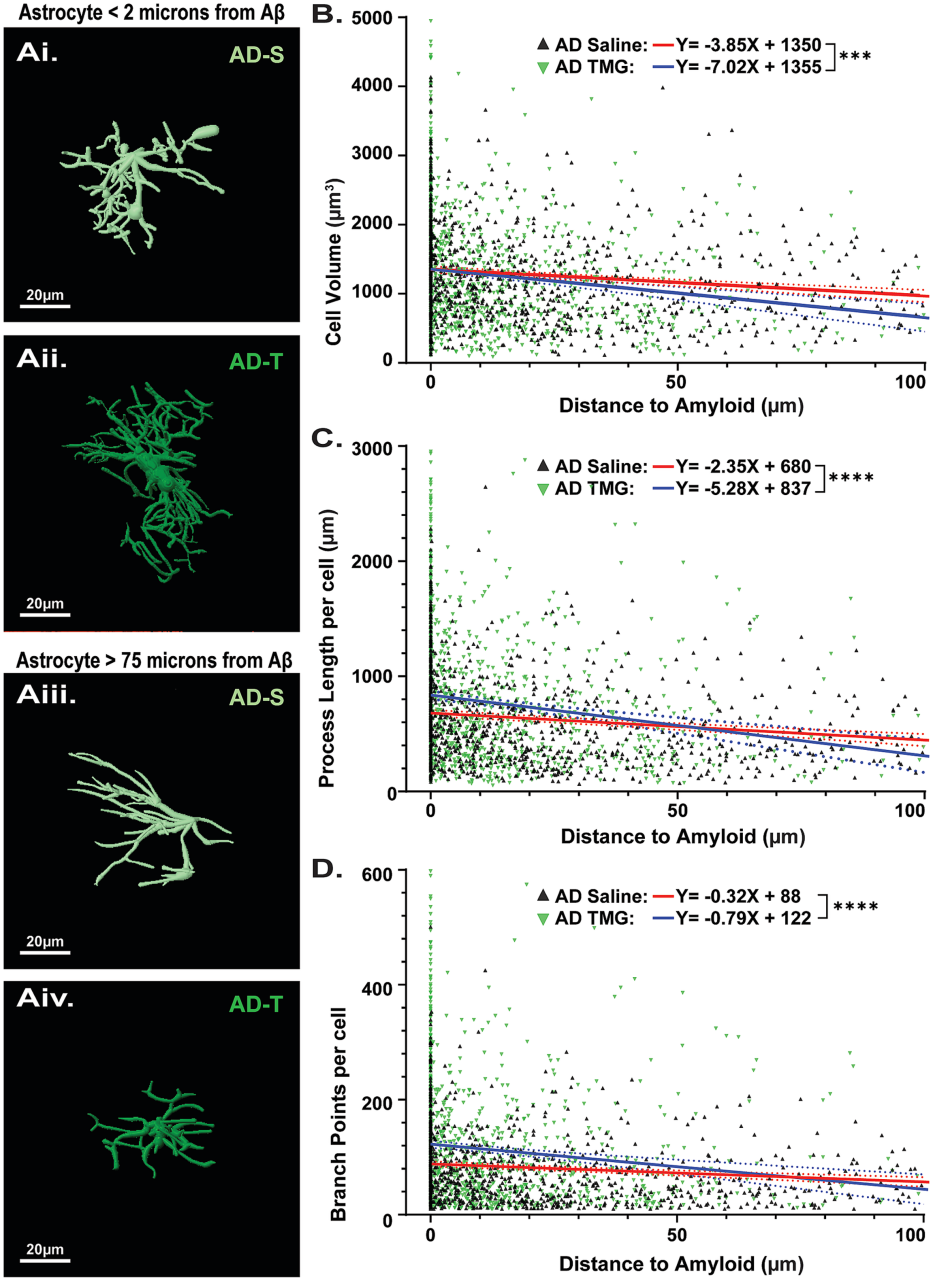
Pharmacologically increasing O-GlcNAc modulates astrocyte complexity based on proximity to Aβ plaques. **(Ai,Aii)** Reconstructed images of astrocytes in TgF344-AD rats located less than 2 μm from an Aβ plaque. **(Aiii,Aiv)** Reconstructed images of astrocytes located at least 75 μm from an Aβ plaque, scale bars = 20 μm. **(B)** Linear regression showing relationship between cell volume and distance to the nearest Aβ plaque (Saline (red line): y = −3.845X + 1,351 vs. TMG (black line) y = −7.015X + 1,355; *p* = 0.0095). **(C)** Linear regression showing relationship between total cell process length and distance to the nearest Aβ plaque (Saline: y = −2.3458X + 681.1 vs. TMG y = −5.278X + 837.2; *p* = 0.0004). **(D)** Linear regression showing relationship between total cell branch points and distance to the nearest Aβ plaque (Saline: y = −0.3116X + 88.13 vs. TMG y = −0.7851X + 122.8; *p* = 0.0009). Across all parameters examined, analyses reveal significantly different linear models between saline and TMG-treated animals, indicating that TMG treatment differentially impacts the relationship between astrocyte complexity and distance to Aβ. Regression plots for linear regressions in Supplementary Figure 5.

Previous studies in transgenic AD mouse models (Chatterjee et al., 2023; Gomez-Arboledas et al., 2018) and postmortem human tissue from AD patients (Bouvier et al., 2016; Wegiel et al., 2000) have determined that astrocyte morphology is more complex near Aβ plaques. Therefore, we next wanted to determine whether the complexity of astrocytes was dependent on their proximity to Aβ plaques and whether they are differentially modulated by increasing O-GlcNAcylation (Figure 7). To evaluate astrocyte morphology with respect to Aβ plaque distance, separate linear regression models were fit for each condition (AD saline vs. AD TMG). Parameters of constructed models (i.e., slope, y-intercept) were then tested to see if constructed models differed significantly between treatment groups. Here, we find that significantly different linear models characterize the relationship between Aβ plaque distance and astrocyte reactivity across all parameters investigated. Indeed, we confirmed that astrocytes near Aβ plaques have larger cell volumes, longer process lengths, and more branch points compared to those located more distally to plaques, regardless of treatment. (Figure 7). Surprisingly, increasing O-GlcNAc further increases cell volume (Figure 7B; saline: y = −3.845X + 1,351 vs. TMG y = −7.015X + 1,355; *p* = 0.0095), process length (Figure 7C; saline: y = −2.3458X + 681.1 vs. TMG y = −5.278X + 837.2; *p* = 0.0004) and number of branch points (Figure 7D; saline: y = −0.3116X + 88.13 vs. TMG y = −-0.7851X + 122.8; *p* = 0.0009) of astrocytes close to plaques (roughly 0 to 30 microns away from the nearest plaque) compared to astrocytes of similar proximity to plaques in saline-treated TgF344-AD rats. Also surprising is that the opposite is true for the astrocytes located further from plaques (roughly 50–75 microns away from the nearest plaque), where astrocytes in TMG-treated TgF344-AD rats are smaller and less complex than those astrocytes in saline-treated TgF344-AD rats of similar distance from plaques.

### 3.7 Microglia in TgF344-AD rats are increased, more reactive, and are resistant to O-GlcNAc modulation

**Figure 8:**
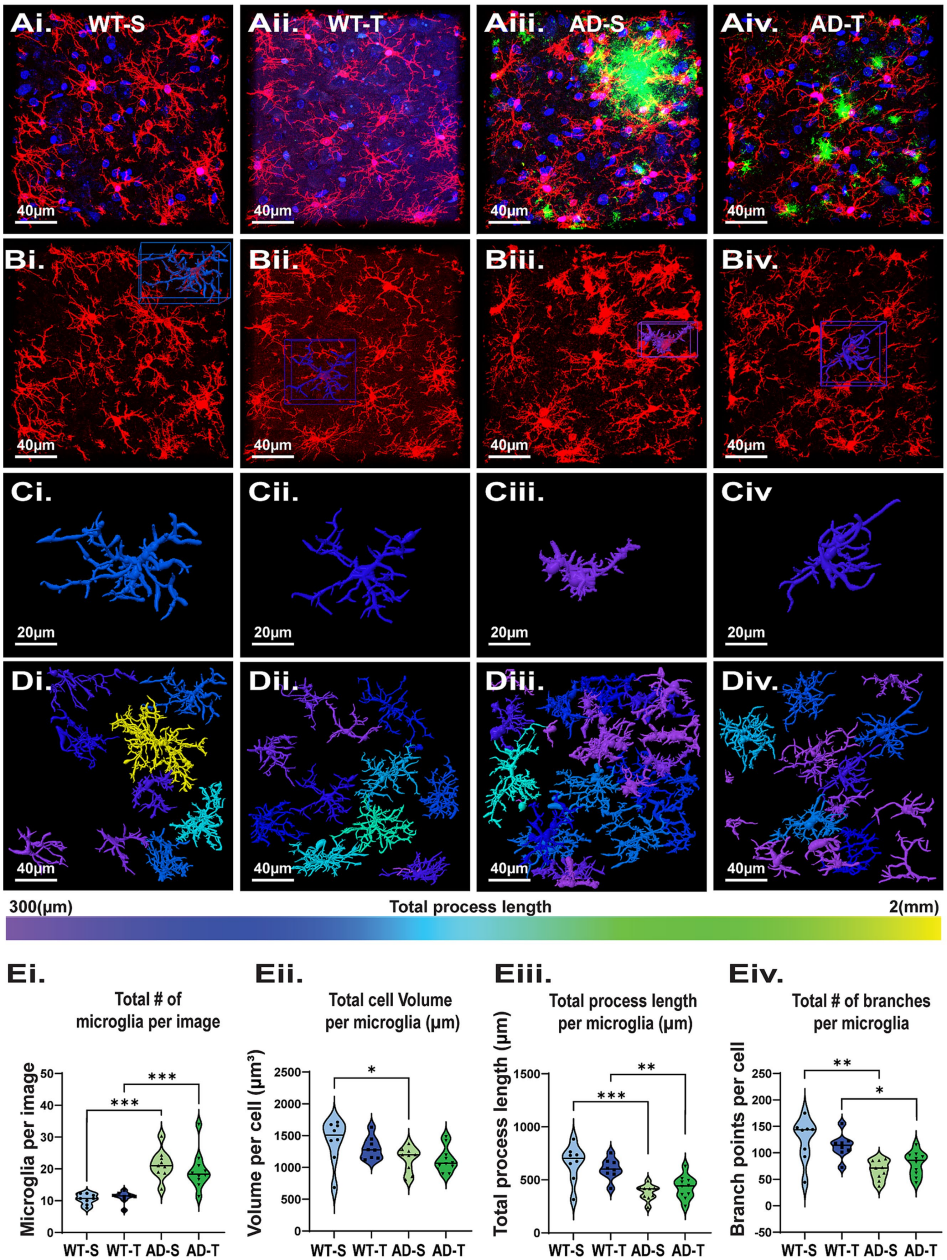
Increasing O-GlcNAc has no significant effect on microglia morphology. **(Ai–Div)** Representative images of microglia from all 4 experimental groups with morphological reconstruction using Arivis. **(Ai–Aiv)** Dapi (blue), Iba-1 (red), Aβ (green), scale bar = 40 μm. **(Bi–Biv)** Images display only Iba-1 staining; scale bar = 40 μm. **(Ci–Civ)** Examples of individual reconstructed microglia. Image color based on total process length of that cell, scale bar = 20 μm. **(Di–Div)** Full image Arivis reconstruction; cell color based on total process length shows an increased number of microglia with shorter total process lengths in TgF344-AD rats (i.e., no yellow). **(Ei–Eiv)** Graphs quantifying morphology attributes of microglia in the hilus. Each dot represents 1 rat with an average of 3 images per rat (total number of microglia analyzed: 1686 total cells, with a breakdown of: WT-S: 252; WT-T: 267; AD-S: 576; AD-T: 591); *N* = 8–10 rats per group. **(Ei)** Significant effect of genotype on total number of microglia with TgF344-AD rats having increased number with no effect of treatment [significant genotype effect: F (1,31)=45.322, *p* < 0.001; no treatment effect: F (1,31)=0.398, *p* = 0.533]; pairwise comparisons show a significant difference between saline-treated WT and TgF344-AD rats (*p* < 0.001) and TMG-treated WT and TgF344-AD rats (*p* < 0.001). **(Eii)** A significant effect of genotype with microglia in TgF344-AD rats having a significantly smaller total volume with no effect of treatment [significant genotype effect: F (1,31)=8.535, *p* = 0.006; no treatment effect: F (1,31)=0.562, *p* = 0.459]; pairwise comparisons show a significant difference between saline-treated WT and TgF344-AD rats (*p* < 0.001). **(Eiii)** A significant effect of genotype with microglia in TgF344-AD rats having a significant decrease in total process length per cell with no effect of treatment [significant genotype effect F (1,31)=31.770, *p* < 0.001; no treatment effect: F (1,31)=0.006, *p* = 0.938]; pairwise comparisons show a significant difference between both saline-treated rats WT and TgF344-AD rats (*p* < 0.001), and TMG-treated WT and AD rats (*p* = 0.004). **(Eiv)** A significant effect of genotype with microglia in TgF344-AD rats having a significant decrease in total branches per cell with no effect of treatment [significant genotype effect: F (1,31)=24.074, *p* < 0.001; no treatment effect: F (1,31)=0.002, *p* = 0.965]; pairwise comparisons show a significant difference between both saline-treated WT and TgF344-AD rats (*p* < 0.001), and TMG-treated WT andTgF344-AD rats (*p* = 0.015).

In TgF344-AD rats, microglia are also reactive, indicated by an increase in Iba-1 staining intensity starting at 6 months of age (Cohen et al., 2013; Rorabaugh et al., 2017). To further characterize these cells, we used IHC staining and confocal imaging and performed a detailed analysis of microglia morphology in the hilus of saline- and TMG-treated TgF344-AD rats compared to saline and TMG-treated WT rats (Figures 8Ai–Div). Although we only found a significant increase of Iba-1 protein in Western blot analysis of TMG-treated WT and TgF344-AD rats (Figure 5B), we found a significant increase in the total number of microglia in TgF344-AD rats compared to WT (*p* < 0.001; Figure 8Ei) in both saline (*p* < 0.001) and TMG-treated groups (*p* < 0.001), with no interaction (*p* = 0.533). This again indicates that measuring proteins like GFAP and Iba-1 can provide useful information, but it does not provide an accurate picture of what is happening to the morphological restructuring of the glial cells. This finding is in contrast to our finding with astrocytes, which are decreased in number in TgF344-AD rats compared to WT (Figure 6). Furthermore, we found a significant genotype difference in total microglia volume with decreased volume in TgF344-AD rats vs. WT (*p* = 0.006; Figure 8 Eii), but this difference was limited to the saline-treated group (*p* = 0.018), and there was no interaction with treatment (*p* = 0.459). Consistent with this, we found a significant effect of genotype (*p* < 0.001) on total process length, with decreased length in TgF344-AD rats compared to WT in both the saline (*p* < 0.001) and TMG-treated groups (*p* = 0.004; Figure 8Eiii), but with no treatment interaction (*p* = 0.938). We observed a similar finding with branch point numbers (Figure 8Eiv), with decreased branching in TgF344-AD rats vs. WT (*p* < 0.001) with both saline (p < 0.001) and TMG-treated groups (*p* = 0.015), and with no treatment interaction (*p* = 0.965). Overall, microglia were less complex in TgF344-AD rats compared to WT, although the total number was increased, consistent with heightened inflammation. Importantly, no effect on microglia morphology was detected as a result of increased O-GlcNAcylation, in contrast to our findings for astrocytes (Figures 6, 7).

### 3.8 Proximity to aβ plaques does not impact microglia morphology, regardless of thiamet-G treatment

**Figure 9:**
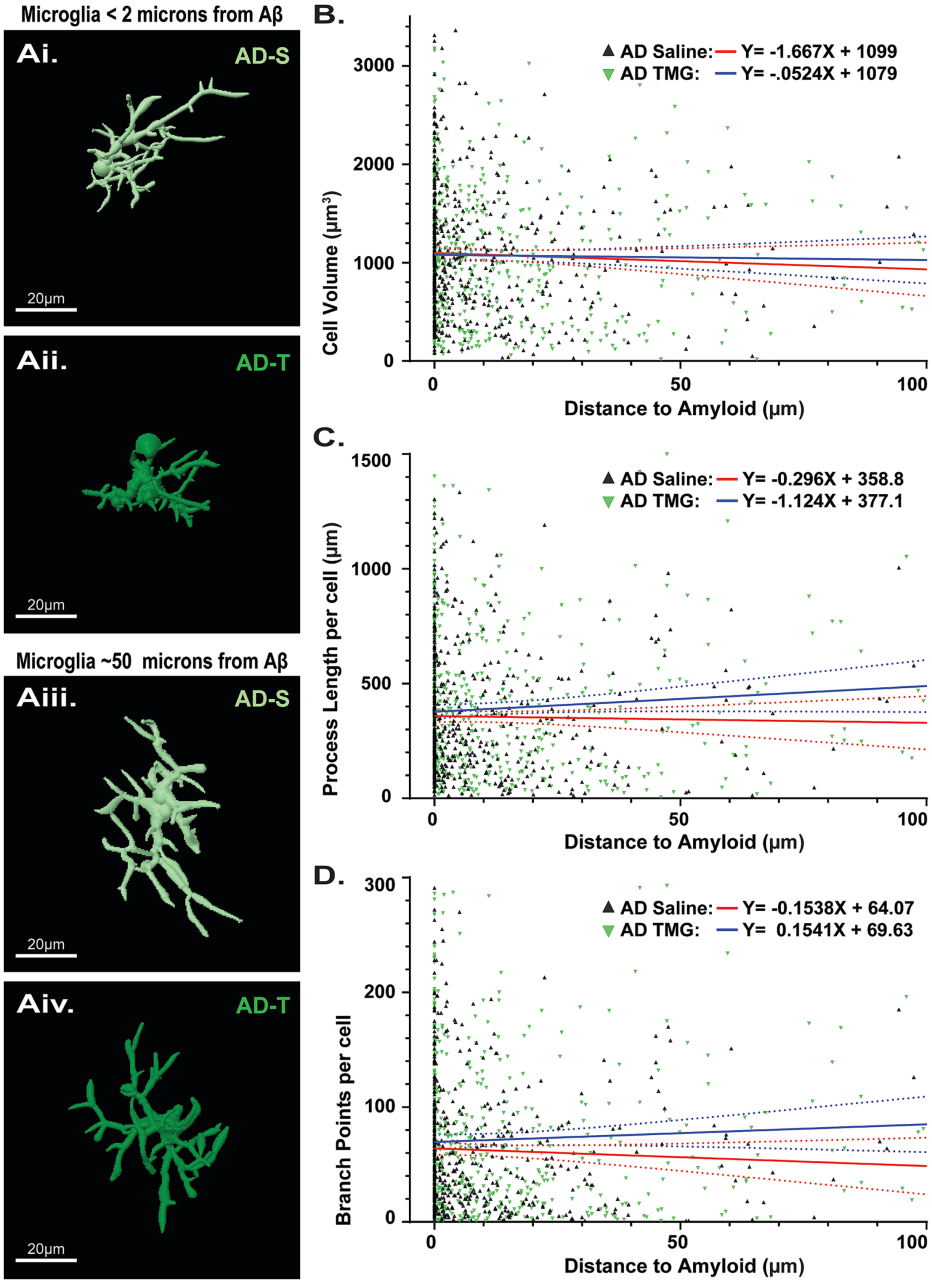
Pharmacologically increasing O-GlcNAc does not affect microglia morphology regardless of proximity to Aβ. **(Ai,Aii)** Reconstructed images of microglia in TgF344-AD rats located less than 2 μm from an Aβ plaque. **(Aiii,Aiv)** Reconstructed images of microglia in TgF344-AD rats located at least 50 μm from an Aβ plaque. Scale bars = 20 μm. **(B)** Linear regression showing relationship between cell volume and distance to the nearest Aβ plaque (Saline: y = −1.667X + 1,099 vs. TMG y = −0.5238X + 1,079; *p* = 0.5711). **(C)** Linear regression showing relationship between total cell process length and distance to the nearest Aβ plaque (Saline: y = −0.2960X + 358.8 vs. TMG y = −1.124X + 377.1; *p* = 0.1202). **(D)** Linear regression showing relationship between total cell branch points and distance to the nearest Aβ plaque (Saline: y = −0.1538X + 64.07 vs. TMG y = 0.1541X + 69.63; *p* = 0.1135). Across all parameters examined, analyses reveal no difference in linear models between saline and TMG TgF344-AD rats, indicating that TMG treatment does not differentially impact the relationship between astrocyte complexity and distance to Aβ. Regression plots for linear regressions in Supplementary Figure 6.

Similar to our analysis with astrocytes, we assessed microglia morphology with respect to proximity to Aβ plaques, and whether increasing O-GlcNAc may affect them, in case we were inadvertently overlooking a finding by comparing averages in the above analysis. In contrast to our findings with astrocytes (Figure 7), there were no significant differences in microglia morphology located proximal (Figures 9Aiii, Aiv) versus distal (Figures 9Ai, Aii) from plaques as indicated by non-significant linear relationships across cell volume (saline: *p* = 0.266; TMG: *p* = 0.699; Figure 9B), process length (saline: *p* = 0.645; TMG: *p* = 0.081; Figure 9C), or branch points (Saline: *p* = 0.259; TMG: *p* = 0.263; Figure 9D). We likewise did not find significant differences in the relationships between treatment groups across cell volume (saline: y = −1.667X + 1,099 vs. TMG y = −0.5238X + 1,079; *p* = 0.5711; Figure 9B), process length (saline: y = −0.2960X + 358.8 vs. TMG y = −1.124X + 377.1; *p* = 0.1202; Figure 9C), or branch points (saline: y = −0.1538X + 64.07 vs. TMG y = 0.1541X + 69.63; *p* = 0.1135; Figure 9D). These findings indicate that microglia morphology is not modulated by increasing O-GlcNAcylation.

### 3.9 Increasing O-GlcNAcylation decreases the number of dystrophic NA axons in Tg-F344-AD rats

**Figure 10:**
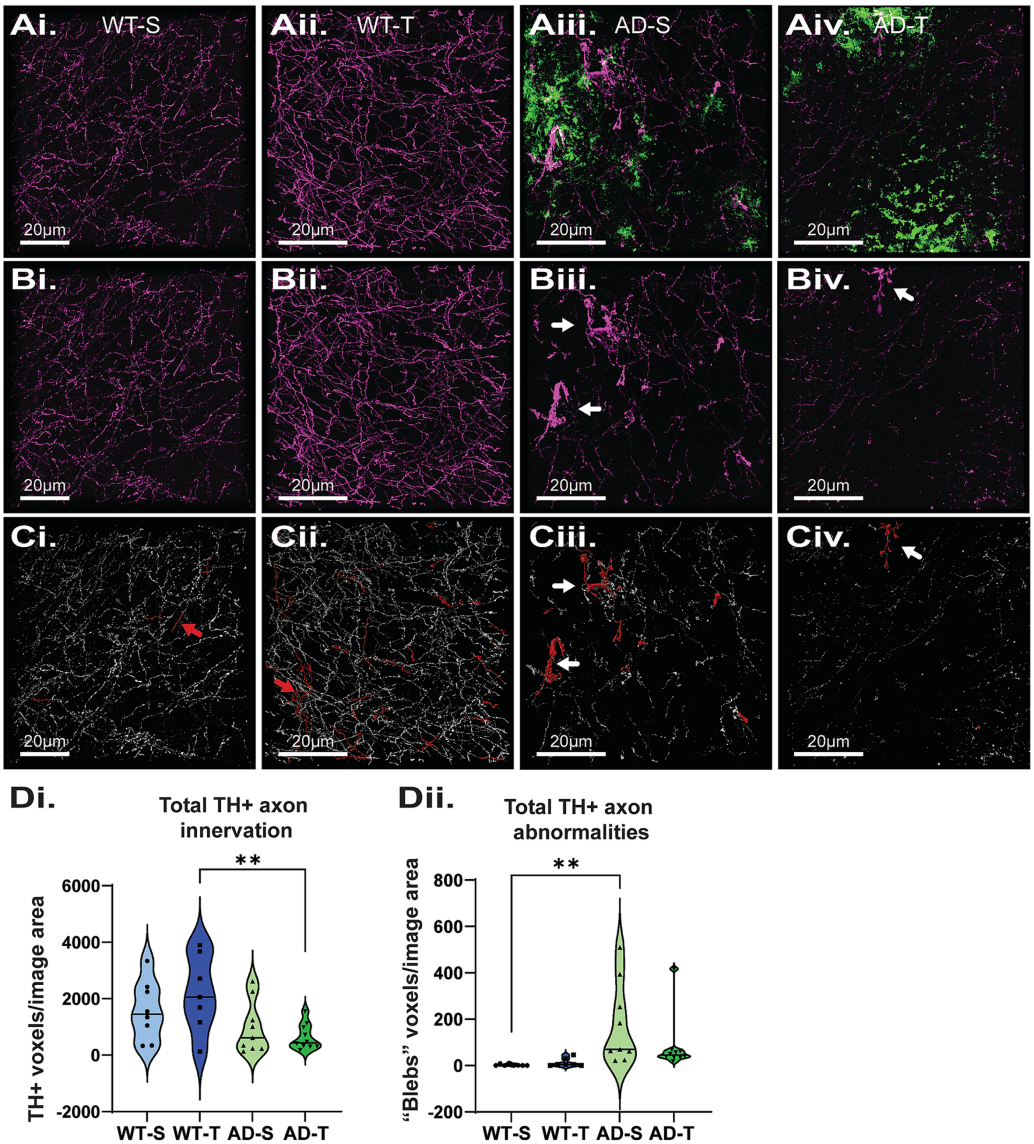
Increasing O-GlcNAc does not lessen the loss of NA axons in the hilus of the DG. **(Ai–Aiv)** Representative images of tyrosine hydroxylase (TH; magenta) and Aβ (green) staining. **(Bi–Biv)** Images displaying only TH staining to facilitate viewing of dystrophic axons (white arrows) in TgF344-AD rats. **(Ci–Civ)** Representative image of TH quantification (white axons), axonal abnormalities (red axons), red arrows indicate abnormalities that were correctly flagged and removed from analysis **(Ci,Cii)** scale bars = 20 μm. **(Di)** A significant effect of genotype with TgF344-AD rats having a significant decrease in total TH axon innervation in the hilus of the DG with no effect of treatment [significant genotype effect: F (1,29)=10.176, *p* = 0.003; no effect of treatment: F (1,29)=0.191, *p* = 0.665; pairwise comparisons show a significant difference between TMG-treated WT and TgF344-AD rats (*p* = 0.004); *N* = 8–10 rats per group, 6 images per rat]. **(Dii)** A significant effect of genotype with TgF344-AD rats having an increase in total dystrophic, torturous and blebbed TH axons with no effect of treatments [significant genotype effect: F (1,29)=10.697, *p* = 0.003; no effect of treatment: F (1,29)=0.409, *p* = 0.527; and no overall interaction: F (1,29)=0.793 *p* = 0.380]. Corrected pairwise comparisons show a significant difference between saline-treated WT and TgF344-AD rats (*p* = 0.006).

A previous study reported no loss of NA axons in 6-month TgF344-AD rats but significant loss at 16 months (Rorabaugh et al., 2017). Subsequently, we reported significant loss of locus coeruleus NA axons in the DG of 6- and 12-month-old TgF344-AD rats compared to WT (Goodman et al., 2021), but interestingly, there was no genotype difference at 9 months, perhaps due to compensatory sprouting. In human AD, evidence of NA axon sprouting in the hippocampus and compensatory changes have been reported (Szot et al., 2007; Szot et al., 2000), along with TH + axons with distinct torturous morphology (Booze et al., 1993). To determine if increasing O-GlcNAcylation between 6 and 9 months of age can protect against loss of NA axons in TgF344-AD rats, we used tyrosine hydroxylase (TH) staining as a proxy for NA innervation and imaged TH + axons in the hilus (Figures 10AI–Civ). We found a genotype difference in TH + staining (*p* = 0.003) and pair-wise comparisons revealed that TMG-treated TgF344-AD rats have less staining compared to TMG-treated WT rats (*p* = 0.004), although there were no differences in the saline-treated groups (*p* = 0.201; Figures 10Di). Importantly, we noted the presence of tortuous, dystrophic TH + axons in both the saline- and TMG-treated TgF344-AD groups (Figures 10Bii–Civ, white arrows), similar to what has been reported in postmortem human AD tissue. To determine if increasing O-GlcNAc improved the morphology, and presumably the health, of NA axons, we quantified the dystrophic “blebby” axons by using size exclusion to separate unusually large axons (above ~250 voxels), before manual confirmation to add or remove objects of interest (Figures 10Ci,Cii, red arrows indicate “false flags”). Although a difference did not bear out in the statistical analysis (*p* = 0.265; Figures 10Dii), visual inspection of TH + axons in the TMG-treated TgF344-AD groups revealed fewer blebby axons. An improvement is indicated in that the TMG-treated TgF344-AD was not significantly different from either the saline-treated TgF344-AD group or either of the WT groups, suggesting that O-GlcNAc is inducing a slight beneficial change in axon morphology in TgF344-AD rats.

## 4 Discussion

This study was the first to investigate the impact of prolonged, pharmacologically induced increase in protein O-GlcNAcylation on morphological complexity of astrocytes and microglia and the density of NA innervation in either control animals or in an experimental model of AD. We specifically focused on these pathological changes in the DG given our previous findings of altered synaptic function at excitatory synapses at MPP-DGC by 6 months of age in TgF344-AD rats (Smith and McMahon, 2018; Goodman et al., 2021; Smith et al., 2022) as well as significant accumulation of Ab, reactive astrocytes and microglia, and degeneration of NA axons reported by us and others (Goodman et al., 2021; Cohen et al., 2013; Rorabaugh et al., 2017; Chaney et al., 2021).

Preclinical data showing beneficial effects of OGA inhibition on memory performance in mouse models of tauopathy and AD has led to significant clinical interest in OGA inhibitors as a potential treatment for neurodegenerative disorders in patients (Yuzwa et al., 2014; Bartolome-Nebreda et al., 2021; Graham et al., 2014; Hastings et al., 2017; Selnick et al., 2019; Permanne et al., 2022; Wheatley et al., 2019). However, the mechanisms underlying the beneficial effects are not fully understood. Here, we tested the hypothesis that globally increasing O-GlcNAcylation in TgF344-AD rats using the OGA inhibitor TMG for 3 months, beginning at 6 months of age, would reduce Aβ plaques, decrease glial cell reactivity, and lessen NA axon loss in the hilus of the DG measured at 9 months of age. This hypothesis was based upon previous reports showing decreased amyloidogenic processing of APP following pharmacologically or genetically increasing O-GlcNAc in transgenic mouse models (Kim et al., 2013; Yuzwa et al., 2014; Park et al., 2021; Borghgraef et al., 2013). Recent literature has shown that pharmacologically increasing O-GlcNAc decreases astrocyte reactivity and GFAP mRNA in control mice (Dong et al., 2023; Bell et al., 2025) and genetically increasing O-GlcNAc in 5XFAD mice decreases GFAP protein expression and astrocyte reactivity (Dong et al., 2023). Furthermore, because increasing O-GlcNAc can decrease the accumulation of both Aβ and pTau, it might protect NA axons from degeneration.

### 4.1 TMG significantly increases O-GlcNAc with a cumulative effect of multiple injections

While only a few studies currently exist, increasing O-GlcNAcylation has been shown to improve memory performance in transgenic mice with Tau or APP mutations (Kim et al., 2013; Yuzwa et al., 2014). The molecular mechanisms driving this beneficial effect are not fully understood but may be related to the ability of O-GlcNAc to enhance non-amyloidogenic APP processing, decrease hyperphosphorylation of Tau as previously mentioned (Kim et al., 2013; Yuzwa et al., 2014; Bartolome-Nebreda et al., 2021; Graham et al., 2014; Dong et al., 2023; Banerjee et al., 2016; Wheatley et al., 2019), regulation of neuronal circuits (Stewart et al., 2020; Taylor et al., 2014; Park et al., 2021; Wheatley et al., 2019; Phillips et al., 2024), and/or potentially by decreasing glial cell reactivity (Dong et al., 2023; Banerjee et al., 2016; Bell et al., 2025; Park et al., 2021; Park et al., 2021; Baudoin and Issad, 2014). Notably, these studies have employed high doses of OGA inhibitors (up to 500 mg/kg in daily drinking water) for extended periods (5 + months) to achieve beneficial effects (Yuzwa et al., 2014; Wang et al., 2020). Here, we used a more modest dose of TMG (10 mg/kg) that we have shown here and in our previous studies to cause robust increases in protein O-GlcNAcylation in the hippocampus (Taylor et al., 2014) and decrease epileptic activity recorded *in vivo* (Stewart et al., 2020; Stewart et al., 2017). We have also reported that this dose can acutely impair some forms of learning and memory (Taylor et al., 2014). To permit the fluctuating nature of O-GlcNAcylation that occurs in vivo (Chatham et al., 2021; Hart et al., 2007; Liu et al., 2024), we increased O-GlcNAcylation using a 3-times-per-week treatment protocol to allow O-GlcNAc levels to vary to minimize adverse effects that can occur as a result of chronically elevated O-GlcNAcylation (Karunakaran and Jeoung, 2010; Umapathi et al., 2021). Little information exists in the published literature about the time course of the increase in O-GlcNAc in the hippocampus following systemic administration of OGA inhibitors, which is important given these drugs are being used in AD clinical trials (Bartolome-Nebreda et al., 2021; Selnick et al., 2019; Permanne et al., 2022). We find that following a single injection of TMG, O-GlcNAc levels return to baseline by 24 h post-injection. However, O-GlcNAcylation remains elevated for 48 h post-injection following a series of 3 injections delivered at a 48 h interval, indicating an accumulative effect. This, along with previous data, led us to try a lower dose of TMG to increase O-GlcNAc, hopefully without incurring the detrimental effects of chronically increased O-GlcNAc seen in several diseases (Karunakaran and Jeoung, 2010; Umapathi et al., 2021; Dias and Hart, 2007). Importantly, we confirmed that O-GlcNAcylation remains elevated in the DG, as well as in CA3 and CA1 (Supplementary Figure 1), following a 3-month treatment, indicating that OGT remains functional and has not been downregulated to offset the chronic inhibition of OGA. It is unknown how O-GlcNAc levels between acutely injected animals vs. chronically injected animals differ, and it is also unknown how long O-GlcNAcylation will remain increased following termination of chronic OGA inhibition, as in the 3-month treatment protocol used here.

### 4.2 Increasing O-GlcNAc at a time when aβ accumulation is significant does not prevent further plaque accumulation

In our experiments, we investigated whether increasing O-GlcNAc could slow further Aβ accumulation once pathology had already begun. In contrast to findings in transgenic AD mice reporting decreased amyloidogenic APP processing under conditions of increased O-GlcNAcylation (Jacobsen and Iverfeldt, 2011; Kim et al., 2013; Yuzwa et al., 2014; Bell et al., 2025; Park et al., 2021), we found no detectable difference in Aβ 15 kDa protein in Western blot analysis or in total plaque volume in IHC staining in the hilus from TMG vs. saline-treated TgF344-AD rats using the specific dose and treatment schedule we employed. Potentially beginning treatment earlier in the disease process, before Aβ accumulation begins, or use of a higher TMG dose may be beneficial in decreasing Aβ accumulation. This awaits future study.

### 4.3 Genotype difference in Iba-1 expression between the thiamet-G treated groups, but no genotype or treatment effect on GFAP protein expression

Despite previous reports of reactive astrocytes and microglia by 6–8 months in TgF344-AD rats via anti-GFAP or anti-Iba-1 IHC (Cohen et al., 2013; Rorabaugh et al., 2017), and although Disterhoft et al., add year has demonstrated increases in GFAP protein starting at 6–8 months assessed via mass spectroscopy in bulk CA1 homogenate (Bac et al., 2023), we observed no detectable difference in GFAP protein levels in DG measured in Western blot across genotype or treatment. We suspect changes in protein level may be masked by decreases in the total number of GFAP-positive astrocytes, even though those that remain exhibit significantly increased total cell volume. In contrast to our findings with GFAP expression, we found a significant increase in Iba-1 expression with elevated levels in the DG of TgF344-AD rats compared to WT, but this was specific to TMG-treated groups, with no significant differences in Iba-1 expression in the saline-treated groups. We did observe a significant increase in GFAP and Iba-1 proteins in the CA3 homogenate of saline-injected TgF344-AD rats compared to saline WT (Supplementary Figure 1), and we suspect even greater morphological differences in glial populations. This awaits future study. To date, no study has used Western blot to quantify GFAP or Iba-1 expression in TgF344-AD rats at any age.

### 4.4 Increasing O-GlcNAc further exacerbates astrocyte reactivity in TgF344-AD

Our finding of fewer astrocytes in TgF344-AD rats compared to WT, regardless of treatment, was unexpected. A previous study in 7–8-month TgF344-AD rats found no change in the number of astrocytes in the hippocampus, although they did find a significant increase in the prefrontal cortex (Futacsi et al., 2025). The decrease in astrocyte number in this study was offset by an overall larger total astrocyte cell volume in TgF344-AD rats, suggesting that individual astrocytes expand to cover a greater region. This is consistent with literature showing variable morphological changes that are highly associated with various reactive states (Sofroniew, 2020; Pekny and Pekna, 2014; Sofroniew, 2014; Burda et al., 2016). With respect to morphological complexity, we found no significant differences when statistically comparing any measure of astrocyte morphology between saline- and TMG-treated WT rats. However, inspection of the violin plots seems to suggest that astrocytes in TMG-treated WT rats have less variability, or a more narrow distribution of process length and number of branches compared to saline-treated WT rats, implying that increasing O-GlcNAc makes astrocyte morphology, and perhaps function, more homogenous. Future studies are needed to fully determine if O-GlcNAc impacts astrocyte function.

### 4.5 Thiamet-G affects astrocyte cell properties based on proximity to aβ

A surprising finding was the selective effect of increasing O-GlcNAc on astrocyte complexity in TgF344-AD rats with no effect on microglia. While we hypothesized that increasing O-GlcNAc would reduce astrocyte reactivity, we observed the complete opposite. Total astrocyte process length and number of branches were significantly increased in TMG- vs. saline-treated TgF344-AD rats, indicating reactivity was further heightened by increasing O-GlcNAcylation. These surprising findings suggest a sort of enhanced function induced by increasing O-GlcNAc on astrocyte complexity in AD, a mechanism that is absent under healthy conditions in WT rats. The functional impact of this effect is currently unknown, but it is attractive to speculate that O-GlcNAc is enhancing astrocyte reactivity as a further defense against the damaging effects of Aβ accumulation. This concept is supported by our analysis of morphological complexity when the astrocytes are in close proximity to Aβ plaques versus at distal locations in TMG vs. saline-treated TgF344-AD rats. Importantly, it has been appreciated for a couple of decades that astrocytes near Aβ plaques are more reactive than those at distal sites (Chen et al., 2025; Sofroniew, 2014; Pekny et al., 2014). Unexpectedly, we found that TMG treatment differentially affected astrocyte characteristics based on distance to Aβ plaques, with astrocytes near plaques being larger and more complex than their counterparts at the same distance in saline-treated TgF344-AD rats, while astrocytes more distally located to Aβ plaques are smaller and less complex compared to astrocytes at the same distance from Aβ plaques in their saline-treated counterparts. Our data suggest that astrocytes may form a protective barrier around amyloid plaques, demarcating the area for Aβ degradation, phagocytosis, and a local inflammatory reaction. This would predict that plaques that are not insulated by astrocytes have a greater toxic effect on surrounding brain tissue. These findings further indicate that TMG is specifically increasing astrocyte complexity near Aβ plaques, possibly to aid in sequestering Aβ plaques to limit damage to surrounding tissue by forming a protective border around plaques (Sofroniew, 2015; Sofroniew, 2014), while decreasing their complexity further away, possibly preventing the pro-inflammatory and cytotoxic cascades that are induced when glial cells are exposed to Aβ (Itagaki et al., 1989; Li et al., 2011). However, further investigations are required to confirm and characterize the neuroprotective role of astrocytes in response to Aβ plaque formation (Mathur et al., 2015) and, in particular, how increasing O-GlcNAcylation modulates astrocyte function in addition to morphology during progressive AD pathology.

### 4.6 Thiamet-G does not reduce microglia reactivity observed in TgF344-AD rats

Microglia are reactive in TgF344-AD rats starting at 6 months of age (Cohen et al., 2013; Rorabaugh et al., 2017), and our findings confirm this in the DG at 9 months of age. Similar to a recent report in TgF344-AD rats (Futacsi et al., 2025), we observed a significant increase in the total number of microglia in TgF344-AD rats compared to WT, and there was no effect of treatment. Our additional analysis showed an overall decrease in microglia cell volume, likely a consequence of the significant decrease in processes contributing to the decrease in overall cell volume. Individual cells in TgF344-AD rats had a more ameboid shape compared to WT, consistent with observations of microglia from both human AD patients and various transgenic models (Colonna and Butovsky, 2017; Davalos et al., 2005; Town et al., 2005; Wendimu and Hooks, 2022; Crespo-Garcia et al., 2015). These findings are in direct contrast to our findings with astrocytes, where their number was decreased and volume increased in TgF344-AD rats compared to WT. Also, in contrast to our findings with astrocytes, microglia in TgF344-AD rats were less complex, with significantly shorter total process length and fewer branch points. Interestingly, microglia morphology, regardless of genotype, appeared resistant to increased O-GlcNAc. However, TMG treatment seemed to normalize microglia volume, such that cell volumes were only different in saline-treated WT and TgF344-AD rats. We also found no significant difference in microglia cell properties with respect to distance to Aβ plaques or treatment, indicating microglia are retaining their reactive state, regardless of proximity to amyloid plaques. Although previous research has shown increasing O-GlcNAc suppresses inflammatory responses through microglial-mediated inflammatory regulation in various models of neurodegeneration or brain injury (Park et al., 2021; He et al., 2017; Hwang et al., 2013; Hwang et al., 2010; Wang et al., 2024; Yao et al., 2025; Zheng et al., 2012), many of these studies only saw effect at high doses of glucosamine (direct substrate for making UDP-GlcNAc), or OGA inhibitors. This indicates that while O-GlcNAc has the ability to modulate microglial activity, either the dose used in this study or the specific cellular environment to induce increased O-GlcNAcylation on microglia was lost or not sustained over time in these rats.

### 4.7 Dystrophic, blebbed NA axons are increased in TgF344-AD

While it has long been recognized that AD patients suffer from early locus coeruleus (LC) degeneration (Weinshenker, 2018; Fructuoso et al., 2025; Szot et al., 2006), it has only been in the past few decades that the field has appreciated how this loss exacerbated neurodegeneration and pathological dysfunction, as seen in rodent models with chemogenic LC degeneration (Chalermpalanupap et al., 2018; Iannitelli et al., 2023; Weinshenker et al., 2008; Evans et al., 2024; Kalinin et al., 2007; Pugh et al., 2007). Numerous studies have shown that noradrenaline is involved in complex inflammation signaling cascades, with loss of NA signaling increasing microglia reactivity (Evans et al., 2020; Gonzalez-Prieto et al., 2021) and perturbations to astrocyte activity (Wahis and Holt, 2021; Lim et al., 2021), and glial cell involvement in the degradation of NA axons (Zong et al., 2025; Jing et al., 2021). We previously reported significant loss of NA axons at 6 and 12 months in the DG of male TgF344-AD rats, but a non-significant decrease at 9 months (Goodman et al., 2021). Others have reported decreased NA innervation at 16 months in TgF344-AD rats, but not at 6 months of age (Rorabaugh et al., 2017). The apparent normalization of NA axon density at 9 months in our previous study in male TgF344-AD rats, when there is significant loss at 6 and 12 months, likely indicates a temporary period of axon sprouting, which is believed to also occur in human AD (Booze et al., 1993; Szot et al., 2007). In a previous study, we also reported heightened LTP magnitude at MPP-DGC at 6 months of age in both male and female TgF344-AD rats that was normalized by 9 months, again suggesting compensatory mechanisms at play (Smith and McMahon, 2018). We later reported that the heightened LTP at MPP-DGC synapses in 6-month-old TgF344-AD rats was caused by an increase in the function of *β*-adrenergic receptors (Goodman et al., 2021; Smith et al., 2022). Interestingly, a recent study found a higher density of cholinergic synapses in area CA1 of TgF344-AD rats just prior to the appearance of Aβ plaques, suggesting cholinergic axon sprouting as a compensatory mechanism (Berg et al., 2025).

Here, our results show a genotype effect, with WT animals having a higher NA axon innervation compared to TgF344-AD rats overall; however, pairwise comparisons revealed this was mainly driven by TMG-treated WT rats. During image analysis, we noted the presence of abnormally thickened axons, often organized in clusters, sometimes termed torturous axons, dystrophic axons, or axonal “blebs” in the literature, particularly in post-mortem human brain tissue, though it has been noted in animal models of AD as well (Booze et al., 1993; Powers et al., 1988; Liu et al., 2013; Harrell et al., 2005). We chose to quantify axonal abnormalities and found a significant increase in these abnormalities in TgF344-AD saline-injected injected compared to WT saline rats. This significance is lost in when comparing TMG-treated WT vs. TMG-treated TgF344-AD rats, presumably due to a protection of NA axons and a reduction in blebbed axons in the TMG-treated TgF344-AD rats, which is supported by visual inspection of the images. Potentially, increasing O-GlcNAc may provide protection to NA axons in AD, but more work is needed to determine if this is the case and alternate doses and treatment strategies may be needed.

### 4.8 Limitations

This study has several limitations. We chose a specific TMG dose (10 mg/kg) leaning on our previous studies showing significant hippocampus dependent behavioral and neuronal excitability changes (Stewart et al., 2020; Stewart et al., 2017) and injection schedule of 3 times per week to allow cycling of O-GlcNAc levels determine whether increasing O-GlcNAcylation could slow ensuing AD pathology. We analyzed only the DG, as it is the first region to display synaptic deficits in TgF344-AD rats (Smith et al., 2022). Thus, investigating other treatment schedules and brain regions will expand an understanding of this potential therapeutic strategy. We systematically quantified Aβ plaques via IHC, therefore, the preciseness of the amyloid load may be limited due to the diversity of plaque size, density, and location. Furthermore, by Western blot, we detection of the 15 kDa fragment, and did not investigate potential differences in Aβ monomers, oligomers, protofibrils, and fibrils. These strategies may be prevented a detection of significant differences in Aβ load between TMG and saline-treated TgF344-AD rats. We also acknowledge that while increases in GFAP are common to infer reactivity, the finer processes and plasma membrane of astrocytes extend far beyond the GFAP-stained arbor. Morphological analysis using a method that can visualize the plasma membrane may reveal other morphological changes. At the outset, we attempted to sufficiently power this study; however, the diverse nature of AD pathogenesis, together with the vast heterogeneity of astrocytes and microglia, and TH innervation/abnormalities, interactions between genotype and treatment were underpowered. Furthermore, due to averaging hundreds of heterogeneous astrocytes (or microglia) into one averaged value per animal, we could be missing morphological differences in various populations of cells. These results reflect the overall morphological attributes of glial cells inside the hilus; thus, we could be missing specific changes occurring in these heterogeneous cell populations. Finally, we used only female rats in this study. Previous studies have used both sexes and report limited differences at the ages used in this study (Cohen et al., 2013; Morrone et al., 2020; Bazzigaluppi et al., 2018; Pentkowski et al., 2018).

## 5 Conclusion

The results of this study further highlight the extensive pathological changes in TgF344-AD rats and the complexity of manipulating O-GlcNAc levels in evaluating beneficial mechanisms and therapeutic potential in AD or other neurodegenerative diseases. Our work enhances understanding of astrocyte and microglia morphological complexity, with the surprising finding of selective effects of increasing O-GlcNAc on astrocytes, with microglia being mostly resistant, at least at the TMG dose and treatment schedule used here. The dual beneficial effects of increasing O-GlcNAc on astrocyte morphological complexity, which depend upon distance from Aβ plaques was also unexpected. Detailed morphological complexity has significant advantage over the use of total surface staining intensity in IHC or protein expression levels in Western blot. Future studies investigating the advantage of increasing O-GlcNAc prior to disease onset are needed to inform therapeutic strategies in delaying the onset of AD pathology.

## Data Availability

The raw data supporting the conclusions of this article will be made available by the authors, without undue reservation.

## References

[ref1] (2024). 2024 Alzheimer's disease facts and figures. Alzheimers Dement. 20, 3708–3821. doi: 10.1002/alz.13809, 38689398 PMC11095490

[ref2] Andres-Benito P. Fernandez-Duenas V. Carmona M. Escobar L. A. Torrejon-Escribano B. Aso E. . (2017). Locus coeruleus at asymptomatic early and middle Braak stages of neurofibrillary tangle pathology. Neuropathol. Appl. Neurobiol. 43, 373–392. doi: 10.1111/nan.12386, 28117912

[ref3] Bac B. Hicheri C. Weiss C. Buell A. Vilcek N. Spaeni C. (2023). The TgF344-AD rat: behavioral and proteomic changes associated with aging and protein expression in a transgenic rat model of Alzheimer's disease. Neurobiol. Aging 123, 98–110. doi: 10.1016/j.neurobiolaging.2022.12.015, 36657371 PMC10118906

[ref4] Bachstetter A. D. Van Eldik L. J. Schmitt F. A. Neltner J. H. Ighodaro E. T. Webster S. J. . (2015). Disease-related microglia heterogeneity in the hippocampus of Alzheimer's disease, dementia with Lewy bodies, and hippocampal sclerosis of aging. Acta Neuropathol. Commun. 3:32. doi: 10.1186/s40478-015-0209-z, 26001591 PMC4489160

[ref5] Baldwin K. T. Murai K. K. Khakh B. S. (2024). Astrocyte morphology. Trends Cell Biol. 34, 547–565. doi: 10.1016/j.tcb.2023.09.006, 38180380 PMC11590062

[ref6] Banerjee P. S. Lagerlof O. Hart G. W. (2016). Roles of O-GlcNAc in chronic diseases of aging. Mol. Asp. Med. 51, 1–15. doi: 10.1016/j.mam.2016.05.005, 27259471

[ref7] Bartolome-Nebreda J. M. Trabanco A. A. Velter A. I. Buijnsters P. (2021). O-GlcNAcase inhibitors as potential therapeutics for the treatment of Alzheimer's disease and related tauopathies: analysis of the patent literature. Expert Opin. Ther. Pat. 31, 1117–1154. doi: 10.1080/13543776.2021.1947242, 34176417

[ref8] Baudoin L. Issad T. (2014). O-GlcNAcylation and inflammation: a vast territory to explore. Front Endocrinol (Lausanne) 5:235. doi: 10.3389/fendo.2014.00235, 25620956 PMC4288382

[ref9] Bazzigaluppi P. Beckett T. L. Koletar M. M. Lai A. Y. Joo I. L. Brown M. E. (2018). Early-stage attenuation of phase-amplitude coupling in the hippocampus and medial prefrontal cortex in a transgenic rat model of Alzheimer's disease. J. Neurochem. 144, 669–679. doi: 10.1111/jnc.14136, 28777881

[ref10] Bell M. B. Kane M. S. Ouyang X. Young M. E. Jegga A. G. Chatham J. C. (2025). Brain transcriptome changes associated with an acute increase of protein O-GlcNAcylation and implications for neurodegenerative disease. J. Neurochem. 169:e16302. doi: 10.1111/jnc.16302, 39823370 PMC11741514

[ref11] Berg M. V. D. Heymans L. Toen D. Adhikari M. A. Audekerke J. V. Verschuuren M. . (2025). Partial normalization of hippocampal oscillatory activity during sleep in TgF344-AD rats coincides with increased cholinergic synapses at early-plaque stage of Alzheimer's disease. Acta Neuropathol. Commun. 13:96. doi: 10.1186/s40478-025-02016-w, 40349073 PMC12065161

[ref12] Booze R. M. Mactutus C. F. Gutman C. R. Davis J. N. (1993). Frequency analysis of catecholamine axonal morphology in human brain. Part II. Alzheimer's disease and hippocampal sympathetic ingrowth. J. Neurol. Sci. 119, 110–118. 7902423 10.1016/0022-510x(93)90198-8

[ref13] Borghgraef P. Menuet C. Theunis C. Louis J. V. Devijver H. Maurin H. (2013). Increasing brain protein O-GlcNAc-ylation mitigates breathing defects and mortality of tau.P301L mice. PLoS One 8:e84442. doi: 10.1371/journal.pone.0084442, 24376810 PMC3871570

[ref14] Borst K. Dumas A. A. Prinz M. (2021). Microglia: immune and non-immune functions. Immunity 54, 2194–2208. doi: 10.1016/j.immuni.2021.09.014, 34644556

[ref15] Bouvier D. S. Jones E. V. Quesseveur G. Davoli M. A. T A. F. Quirion R. . (2016). High resolution dissection of reactive glial nets in Alzheimer's disease. Sci. Rep. 6:24544. doi: 10.1038/srep24544 27090093 PMC4835751

[ref16] Braak H. Del Tredici K. (2012). Where, when, and in what form does sporadic Alzheimer's disease begin? Curr. Opin. Neurol. 25, 708–714. doi: 10.1097/WCO.0b013e32835a3432, 23160422

[ref17] Braak H. Thal D. R. Ghebremedhin E. Del Tredici K. (2011). Stages of the pathologic process in Alzheimer disease: age categories from 1 to 100 years. J. Neuropathol. Exp. Neurol. 70, 960–969. doi: 10.1097/NEN.0b013e318232a379, 22002422

[ref18] Brandebura A. N. Paumier A. Onur T. S. Allen N. J. (2023). Astrocyte contribution to dysfunction, risk and progression in neurodegenerative disorders. Nat. Rev. Neurosci. 24, 23–39. doi: 10.1038/s41583-022-00641-1, 36316501 PMC10198620

[ref19] Burda J. E. Bernstein A. M. Sofroniew M. V. (2016). Astrocyte roles in traumatic brain injury. Exp. Neurol. 275, 305–315. doi: 10.1016/j.expneurol.2015.03.020 25828533 PMC4586307

[ref20] Chalermpalanupap T. Schroeder J. P. Rorabaugh J. M. Liles L. C. Lah J. J. Levey A. I. (2018). Locus Coeruleus ablation exacerbates cognitive deficits, neuropathology, and lethality in P301S tau transgenic mice. J. Neurosci. 38, 74–92. doi: 10.1523/JNEUROSCI.1483-17.2017, 29133432 PMC5761438

[ref21] Chaney A. M. Lopez-Picon F. R. Serriere S. Wang R. Bochicchio D. Webb S. D. . (2021). Prodromal neuroinflammatory, cholinergic and metabolite dysfunction detected by PET and MRS in the TgF344-AD transgenic rat model of AD: a collaborative multi-modal study. Theranostics 11, 6644–6667. doi: 10.7150/thno.56059, 34093845 PMC8171096

[ref22] Chatham J. C. Zhang J. Wende A. R. (2021). Role of O-linked N-acetylglucosamine protein modification in cellular (patho)physiology. Physiol. Rev. 101, 427–493. doi: 10.1152/physrev.00043.2019, 32730113 PMC8428922

[ref23] Chatterjee P. Vermunt L. Gordon B. A. Pedrini S. Boonkamp L. Armstrong N. J. . (2023). Plasma glial fibrillary acidic protein in autosomal dominant Alzheimer's disease: associations with Abeta-PET, neurodegeneration, and cognition. Alzheimers Dement. 19, 2790–2804. doi: 10.1002/alz.12879 36576155 PMC10300233

[ref24] Chen J. Xu S. Wang L. Liu X. Liu G. Tan Q. (2025). Refining the interactions between microglia and astrocytes in Alzheimer's disease pathology. Neuroscience 573, 183–197. doi: 10.1016/j.neuroscience.2025.03.033, 40120713

[ref25] Cohen R. M. Rezai-Zadeh K. Weitz T. M. Rentsendorj A. Gate D. Spivak I. . (2013). A transgenic Alzheimer rat with plaques, tau pathology, behavioral impairment, oligomeric abeta, and frank neuronal loss. J. Neurosci. 33, 6245–6256. doi: 10.1523/JNEUROSCI.3672-12.2013 23575824 PMC3720142

[ref26] Colonna M. Butovsky O. (2017). Microglia function in the central nervous system during health and neurodegeneration. Annu. Rev. Immunol. 35, 441–468. doi: 10.1146/annurev-immunol-051116-052358, 28226226 PMC8167938

[ref27] Cornell J. Salinas S. Huang H. Y. Zhou M. (2022). Microglia regulation of synaptic plasticity and learning and memory. Neural Regen. Res. 17, 705–716. doi: 10.4103/1673-5374.322423, 34472455 PMC8530121

[ref28] Crespo-Garcia S. Reichhart N. Hernandez-Matas C. Zabulis X. Kociok N. Brockmann C. (2015). In vivo analysis of the time and spatial activation pattern of microglia in the retina following laser-induced choroidal neovascularization. Exp. Eye Res. 139, 13–21. doi: 10.1016/j.exer.2015.07.012, 26213305

[ref29] Dahl M. J. Kulesza A. Werkle-Bergner M. Mather M. (2023). Declining locus coeruleus-dopaminergic and noradrenergic modulation of long-term memory in aging and Alzheimer's disease. Neurosci. Biobehav. Rev. 153:105358. doi: 10.1016/j.neubiorev.2023.105358, 37597700 PMC10591841

[ref30] Dahl M. J. Mather M. Werkle-Bergner M. Kennedy B. L. Guzman S. Hurth K. (2022). Locus coeruleus integrity is related to tau burden and memory loss in autosomal-dominant Alzheimer's disease. Neurobiol. Aging 112, 39–54. doi: 10.1016/j.neurobiolaging.2021.11.006, 35045380 PMC8976827

[ref31] Davalos D. Grutzendler J. Yang G. Kim J. V. Zuo Y. Jung S. (2005). ATP mediates rapid microglial response to local brain injury in vivo. Nat. Neurosci. 8, 752–758. doi: 10.1038/nn1472, 15895084

[ref32] Davies D. S. Ma J. Jegathees T. Goldsbury C. (2017). Microglia show altered morphology and reduced arborization in human brain during aging and Alzheimer's disease. Brain Pathol. 27, 795–808. doi: 10.1111/bpa.12456, 27862631 PMC8029278

[ref33] De Strooper B. Karran E. (2016). The cellular phase of Alzheimer's disease. Cell 164, 603–615. doi: 10.1016/j.cell.2015.12.056, 26871627

[ref34] Deng Q. Wu C. Parker E. Liu T. C. Duan R. Yang L. (2024). Microglia and astrocytes in Alzheimer's disease: significance and summary of recent advances. Aging Dis. 15, 1537–1564. doi: 10.14336/AD.2023.0907, 37815901 PMC11272214

[ref35] Denton A. R. Mactutus C. F. Lateef A. U. Harrod S. B. Booze R. M. (2021). Chronic SSRI treatment reverses HIV-1 protein-mediated synaptodendritic damage. J. Neuro-Oncol. 27, 403–421. doi: 10.1007/s13365-021-00960-6, 34003469 PMC8504184

[ref36] Denton A. R. Samaranayake S. A. Kirchner K. N. Roscoe R. F. Jr. Berger S. N. Harrod S. B. (2019). Selective monoaminergic and histaminergic circuit dysregulation following long-term HIV-1 protein exposure. J. Neuro-Oncol. 25, 540–550. doi: 10.1007/s13365-019-00754-x, 31102184 PMC6750960

[ref37] Dias W. B. Hart G. W. (2007). O-GlcNAc modification in diabetes and Alzheimer's disease. Mol. BioSyst. 3, 766–772. doi: 10.1039/b704905f, 17940659

[ref38] Doig A. J. (2018). Positive feedback loops in Alzheimer's disease: the Alzheimer's feedback hypothesis. J Alzheimer's Dis 66, 25–36. doi: 10.3233/JAD-180583, 30282364 PMC6484277

[ref39] Dong X. Shu L. Zhang J. Yang X. Cheng X. Zhao X. . (2023). Ogt-mediated O-GlcNAcylation inhibits astrocytes activation through modulating NF-kappaB signaling pathway. J. Neuroinflammation 20:146. doi: 10.1186/s12974-023-02824-8 37349834 PMC10286367

[ref40] Drago F. Lombardi M. Prada I. Gabrielli M. Joshi P. Cojoc D. (2017). ATP modifies the proteome of extracellular vesicles released by microglia and influences their action on astrocytes. Front. Pharmacol. 8:910. doi: 10.3389/fphar.2017.00910, 29321741 PMC5733563

[ref41] Dyer-Reaves K. Goodman A. M. Nelson A. R. McMahon L. L. (2019). Alpha1-adrenergic receptor mediated long-term depression at CA3-CA1 synapses can be induced via accumulation of endogenous norepinephrine and is preserved following noradrenergic denervation. Front. Synaptic Neurosci. 11:27. doi: 10.3389/fnsyn.2019.00027, 31649525 PMC6794465

[ref42] Evans A. K. Ardestani P. M. Yi B. Park H. H. Lam R. K. Shamloo M. (2020). Beta-adrenergic receptor antagonism is proinflammatory and exacerbates neuroinflammation in a mouse model of Alzheimer's disease. Neurobiol. Dis. 146:105089. doi: 10.1016/j.nbd.2020.105089, 32971233 PMC7686098

[ref43] Evans A. K. Park H. H. Woods C. E. Lam R. K. Rijsketic D. R. Xu C. (2024). Impact of noradrenergic inhibition on neuroinflammation and pathophysiology in mouse models of Alzheimer's disease. J. Neuroinflammation 21:322. doi: 10.1186/s12974-024-03306-1, 39696597 PMC11657531

[ref44] Faghihi M. A. Modarresi F. Khalil A. M. Wood D. E. Sahagan B. G. Morgan T. E. (2008). Expression of a noncoding RNA is elevated in Alzheimer's disease and drives rapid feed-forward regulation of beta-secretase. Nat. Med. 14, 723–730. doi: 10.1038/nm1784, 18587408 PMC2826895

[ref45] Festing M. F. Altman D. G. (2002). Guidelines for the design and statistical analysis of experiments using laboratory animals. ILAR J. 43, 244–258. doi: 10.1093/ilar.43.4.244, 12391400

[ref46] Fructuoso M. Vermeiren Y. Boluda S. Stimmer L. Crans R. A. J. Xicota L. (2025). Disease-specific neuropathological alterations of the locus coeruleus in Alzheimer's disease, down syndrome, and Parkinson's disease. Alzheimers Dement. 21:e70262. doi: 10.1002/alz.70262, 40501099 PMC12159339

[ref47] Fu J. Li L. Huo D. Zhi S. Yang R. Yang B. . (2021). Astrocyte-derived TGFbeta1 facilitates blood-brain barrier function via non-canonical hedgehog signaling in brain microvascular endothelial cells. Brain Sci. 11:77. doi: 10.3390/brainsci11010077 33430164 PMC7826596

[ref48] Futacsi A. Rusznak K. Szarka G. Volgyi B. Wiborg O. Czeh B. (2025). Quantification and correlation of amyloid-beta plaque load, glial activation, GABAergic interneuron numbers, and cognitive decline in the young TgF344-AD rat model of Alzheimer's disease. Front. Aging Neurosci. 17:1542229. doi: 10.3389/fnagi.2025.1542229 40013092 PMC11860898

[ref49] Gao V. Suzuki A. Magistretti P. J. Lengacher S. Pollonini G. Steinman M. Q. . (2016). Astrocytic beta2-adrenergic receptors mediate hippocampal long-term memory consolidation. Proc. Natl. Acad. Sci. USA 113, 8526–8531. doi: 10.1073/pnas.1605063113 27402767 PMC4968707

[ref50] Gomez-Arboledas A. Davila J. C. Sanchez-Mejias E. Navarro V. Nunez-Diaz C. Sanchez-Varo R. . (2018). Phagocytic clearance of presynaptic dystrophies by reactive astrocytes in Alzheimer's disease. Glia 66, 637–653. doi: 10.1002/glia.23270, 29178139 PMC5814816

[ref51] Gonzalez-Prieto M. Gutierrez I. L. Garcia-Bueno B. Caso J. R. Leza J. C. Ortega-Hernandez A. . (2021). Microglial CX3CR1 production increases in Alzheimer's disease and is regulated by noradrenaline. Glia 69, 73–90. doi: 10.1002/glia.23885, 32662924

[ref52] Goodman A. M. Langner B. M. Jackson N. Alex C. McMahon L. L. (2021). Heightened hippocampal beta-adrenergic receptor function drives synaptic potentiation and supports learning and memory in the TgF344-AD rat model during prodromal Alzheimer's disease. J. Neurosci. 41, 5747–5761. doi: 10.1523/JNEUROSCI.0119-21.2021 33952633 PMC8244969

[ref53] Graham D. L. Gray A. J. Joyce J. A. Yu D. O'Moore J. Carlson G. A. . (2014). Increased O-GlcNAcylation reduces pathological tau without affecting its normal phosphorylation in a mouse model of tauopathy. Neuropharmacology 79, 307–313. doi: 10.1016/j.neuropharm.2013.11.025, 24326295

[ref54] Griffith L. S. Mathes M. Schmitz B. (1995). Beta-amyloid precursor protein is modified with O-linked N-acetylglucosamine. J. Neurosci. Res. 41, 270–278. 7650762 10.1002/jnr.490410214

[ref55] Grudzien A. Shaw P. Weintraub S. Bigio E. Mash D. C. Mesulam M. M. (2007). Locus coeruleus neurofibrillary degeneration in aging, mild cognitive impairment and early Alzheimer's disease. Neurobiol. Aging 28, 327–335. doi: 10.1016/j.neurobiolaging.2006.02.007, 16574280

[ref56] Habib N. McCabe C. Medina S. Varshavsky M. Kitsberg D. Dvir-Szternfeld R. (2020). Disease-associated astrocytes in Alzheimer's disease and aging. Nat. Neurosci. 23, 701–706. doi: 10.1038/s41593-020-0624-8, 32341542 PMC9262034

[ref57] Harrell L. E. Parsons D. Kolasa K. (2001). Hippocampal sympathetic ingrowth occurs following 192-IgG-Saporin administration. Brain Res. 911, 158–162. doi: 10.1016/S0006-8993(01)02626-9, 11511384

[ref58] Harrell L. E. Parsons D. S. Kolasa K. (2005). The effect of central cholinergic and noradrenergic denervation on hippocampal sympathetic ingrowth and apoptosis-like reactivity in the rat. Brain Res. 1033, 68–77. doi: 10.1016/j.brainres.2004.11.021, 15680341

[ref59] Hart G. W. Housley M. P. Slawson C. (2007). Cycling of O-linked beta-N-acetylglucosamine on nucleocytoplasmic proteins. Nature 446, 1017–1022. doi: 10.1038/nature05815, 17460662

[ref60] Hart G. W. Slawson C. Ramirez-Correa G. Lagerlof O. (2011). Cross talk between O-GlcNAcylation and phosphorylation: roles in signaling, transcription, and chronic disease. Annu. Rev. Biochem. 80, 825–858. doi: 10.1146/annurev-biochem-060608-102511, 21391816 PMC3294376

[ref61] Hastings N. B. Wang X. Song L. Butts B. D. Grotz D. Hargreaves R. (2017). Inhibition of O-GlcNAcase leads to elevation of O-GlcNAc tau and reduction of tauopathy and cerebrospinal fluid tau in rTg4510 mice. Mol. Neurodegener. 12:39. doi: 10.1186/s13024-017-0181-0, 28521765 PMC5437664

[ref62] He Y. Ma X. Li D. Hao J. (2017). Thiamet G mediates neuroprotection in experimental stroke by modulating microglia/macrophage polarization and inhibiting NF-kappaB p65 signaling. J. Cereb. Blood Flow Metab. 37, 2938–2951. doi: 10.1177/0271678X16679671, 27864466 PMC5536801

[ref63] Hwang S. Y. Hwang J. S. Kim S. Y. Han I. O. (2013). O-GlcNAcylation and p50/p105 binding of c-Rel are dynamically regulated by LPS and glucosamine in BV2 microglia cells. Br. J. Pharmacol. 169, 1551–1560. doi: 10.1111/bph.12223, 23646894 PMC3724111

[ref64] Hwang S. Y. Shin J. H. Hwang J. S. Kim S. Y. Shin J. A. Oh E. S. (2010). Glucosamine exerts a neuroprotective effect via suppression of inflammation in rat brain ischemia/reperfusion injury. Glia 58, 1881–1892. doi: 10.1002/glia.21058, 20737476

[ref65] Iannitelli A. F. Kelberman M. A. Lustberg D. J. Korukonda A. McCann K. E. Mulvey B. . (2023). The neurotoxin DSP-4 dysregulates the locus coeruleus-norepinephrine system and recapitulates molecular and behavioral aspects of prodromal neurodegenerative disease. ENeuro 10:ENEURO.0483-22.2022. doi: 10.1523/ENEURO.0483-22.2022, 36635251 PMC9829100

[ref66] Itagaki S. McGeer P. L. Akiyama H. Zhu S. Selkoe D. (1989). Relationship of microglia and astrocytes to amyloid deposits of Alzheimer disease. J. Neuroimmunol. 24, 173–182. 2808689 10.1016/0165-5728(89)90115-x

[ref67] Jacobsen K. T. Iverfeldt K. (2011). O-glcnacylation increases non-amyloidogenic processing of the amyloid-beta precursor protein (APP). Biochem. Biophys. Res. Commun. 404, 882–886. doi: 10.1016/j.bbrc.2010.12.080 21182826

[ref68] Jing L. Hou L. Zhang D. Li S. Ruan Z. Zhang X. (2021). Microglial activation mediates noradrenergic locus Coeruleus neurodegeneration via complement receptor 3 in a rotenone-induced Parkinson's disease mouse model. J. Inflamm. Res. 14, 1341–1356. doi: 10.2147/JIR.S299927, 33859489 PMC8044341

[ref69] Jo M. Kim J. H. Song G. J. Seo M. Hwang E. M. Suk K. (2017). Astrocytic Orosomucoid-2 modulates microglial activation and Neuroinflammation. J. Neurosci. 37, 2878–2894. doi: 10.1523/JNEUROSCI.2534-16.2017, 28193696 PMC6596722

[ref70] Kalinin S. Gavrilyuk V. Polak P. E. Vasser R. Zhao J. Heneka M. T. (2007). Noradrenaline deficiency in brain increases beta-amyloid plaque burden in an animal model of Alzheimer's disease. Neurobiol. Aging 28, 1206–1214. doi: 10.1016/j.neurobiolaging.2006.06.003, 16837104

[ref71] Karunakaran U. Jeoung N. H. (2010). O-GlcNAc modification: friend or foe in diabetic cardiovascular disease. Korean Diabetes J. 34, 211–219. doi: 10.4093/kdj.2010.34.4.211, 20835337 PMC2932889

[ref72] Kelly S. C. He B. Perez S. E. Ginsberg S. D. Mufson E. J. Counts S. E. (2017). Locus coeruleus cellular and molecular pathology during the progression of Alzheimer's disease. Acta Neuropathol. Commun. 5:8. doi: 10.1186/s40478-017-0411-2, 28109312 PMC5251221

[ref73] Kim S. Chun H. Kim Y. Kim Y. Park U. Chu J. . (2024). Astrocytic autophagy plasticity modulates Abeta clearance and cognitive function in Alzheimer's disease. Mol. Neurodegener. 19:55. doi: 10.1186/s13024-024-00740-w 39044253 PMC11267931

[ref74] Kim C. Nam D. W. Park S. Y. Song H. Hong H. S. Boo J. H. . (2013). O-linked beta-N-acetylglucosaminidase inhibitor attenuates beta-amyloid plaque and rescues memory impairment. Neurobiol. Aging 34, 275–285. doi: 10.1016/j.neurobiolaging.2012.03.001 22503002

[ref75] Kraft A. W. Hu X. Yoon H. Yan P. Xiao Q. Wang Y. (2013). Attenuating astrocyte activation accelerates plaque pathogenesis in APP/PS1 mice. FASEB J. 27, 187–198. doi: 10.1096/fj.12-208660, 23038755 PMC3528309

[ref76] Lalo U. Pankratov Y. Parpura V. Verkhratsky A. (2011). Ionotropic receptors in neuronal-astroglial signalling: what is the role of "excitable" molecules in non-excitable cells. Biochim. Biophys. Acta 1813, 992–1002. doi: 10.1016/j.bbamcr.2010.09.007, 20869992

[ref77] Lana D. Ugolini F. Wenk G. L. Giovannini M. G. Zecchi-Orlandini S. Nosi D. (2019). Microglial distribution, branching, and clearance activity in aged rat hippocampus are affected by astrocyte meshwork integrity: evidence of a novel cell-cell interglial interaction. FASEB J. 33, 4007–4020. doi: 10.1096/fj.201801539R, 30496700

[ref78] Lee E. Jung Y. J. Park Y. R. Lim S. Choi Y. J. Lee S. Y. (2022). A distinct astrocyte subtype in the aging mouse brain characterized by impaired protein homeostasis. Nat Aging. 2, 726–741. doi: 10.1038/s43587-022-00257-1, 37118130

[ref79] Leyns C. E. G. Holtzman D. M. (2017). Glial contributions to neurodegeneration in tauopathies. Mol. Neurodegener. 12:50. doi: 10.1186/s13024-017-0192-x, 28662669 PMC5492997

[ref80] Li C. Zhao R. Gao K. Wei Z. Yin M. Y. Lau L. T. . (2011). Astrocytes: implications for neuroinflammatory pathogenesis of Alzheimer's disease. Curr. Alzheimer Res. 8, 67–80. doi: 10.2174/156720511794604543, 21143158

[ref81] Liddelow S. A. Barres B. A. (2017). Reactive astrocytes: production, function, and therapeutic potential. Immunity 46, 957–967. doi: 10.1016/j.immuni.2017.06.006, 28636962

[ref82] Liddelow S. A. Guttenplan K. A. Clarke L. E. Bennett F. C. Bohlen C. J. Schirmer L. (2017). Neurotoxic reactive astrocytes are induced by activated microglia. Nature 541, 481–487. doi: 10.1038/nature21029, 28099414 PMC5404890

[ref83] Liddelow S. A. Olsen M. L. Sofroniew M. V. (2024). Reactive astrocytes and emerging roles in central nervous system (CNS) disorders. Cold Spring Harb. Perspect. Biol. 16:356. doi: 10.1101/cshperspect.a041356, 38316554 PMC11216178

[ref84] Lim E. Y. Ye L. Paukert M. (2021). Potential and realized impact of Astroglia ca(2 +) dynamics on circuit function and behavior. Front. Cell. Neurosci. 15:682888. doi: 10.3389/fncel.2021.682888, 34163330 PMC8215280

[ref85] Lin C. P. Frigerio I. Bol J. Bouwman M. M. A. Wesseling A. J. Dahl M. J. . (2024). Microstructural integrity of the locus coeruleus and its tracts reflect noradrenergic degeneration in Alzheimer's disease and Parkinson's disease. Transl. Neurodegener. 13:9. doi: 10.1186/s40035-024-00400-5, 38336865 PMC10854137

[ref86] Liu K. Aierken A. Liu M. Parhat N. Kong W. Yin X. (2024). The decreased astrocyte-microglia interaction reflects the early characteristics of Alzheimer's disease. iScience. 27:109281. doi: 10.1016/j.isci.2024.109281, 38455972 PMC10918213

[ref87] Liu X. Cai Y. D. Chiu J. C. (2024). Regulation of protein O-GlcNAcylation by circadian, metabolic, and cellular signals. J. Biol. Chem. 300:105616. doi: 10.1016/j.jbc.2023.105616, 38159854 PMC10810748

[ref88] Liu L. Luo S. Zeng L. Wang W. Yuan L. Jian X. (2013). Degenerative alterations in noradrenergic neurons of the locus coeruleus in Alzheimer's disease. Neural Regen. Res. 8, 2249–2255. doi: 10.3969/j.issn.1673-5374.2013.24.004, 25206534 PMC4146034

[ref89] Liu W. Tang Y. Feng J. (2011). Cross talk between activation of microglia and astrocytes in pathological conditions in the central nervous system. Life Sci. 89, 141–146. doi: 10.1016/j.lfs.2011.05.011, 21684291

[ref90] Llorens F. Thune K. Andres-Benito P. Tahir W. Ansoleaga B. Hernandez-Ortega K. . (2017). MicroRNA expression in the locus Coeruleus, entorhinal cortex, and Hippocampus at early and middle stages of Braak neurofibrillary tangle pathology. J. Mol. Neurosci. 63, 206–215. doi: 10.1007/s12031-017-0971-4, 28871468

[ref91] Lochhead J. J. Yang J. Ronaldson P. T. Davis T. P. (2020). Structure, function, and regulation of the blood-brain barrier tight junction in central nervous system disorders. Front. Physiol. 11:914. doi: 10.3389/fphys.2020.00914, 32848858 PMC7424030

[ref92] Lopez-Ortiz A. O. Eyo U. B. (2024). Astrocytes and microglia in the coordination of CNS development and homeostasis. J. Neurochem. 168, 3599–3614. doi: 10.1111/jnc.16006, 37985374 PMC11102936

[ref93] Martinez-Gallego I. Rodriguez-Moreno A. (2023). Adenosine and astrocytes control critical periods of neural plasticity. Neuroscientist 29, 532–537. doi: 10.1177/10738584221126632, 36245418

[ref94] Matejuk A. Ransohoff R. M. (2020). Crosstalk between astrocytes and microglia: an overview. Front. Immunol. 11:1416. doi: 10.3389/fimmu.2020.01416, 32765501 PMC7378357

[ref95] Mathur R. Ince P. G. Minett T. Garwood C. J. Shaw P. J. Matthews F. E. . (2015). A reduced astrocyte response to beta-amyloid plaques in the ageing brain associates with cognitive impairment. PLoS One 10:e0118463. doi: 10.1371/journal.pone.0118463 25707004 PMC4338046

[ref96] Mitchener V. F. T. Thackray M. J. Arancibia-Carcamo I. L. (2025). The glia-immune network: astrocytes and oligodendrocytes as microglial co-ordinators in health and disease. J. Physiol. doi: 10.1113/JP287015 PMC12909661 40492604

[ref97] Morrone C. D. Bazzigaluppi P. Beckett T. L. Hill M. E. Koletar M. M. Stefanovic B. . (2020). Regional differences in Alzheimer's disease pathology confound behavioural rescue after amyloid-beta attenuation. Brain 143, 359–373. doi: 10.1093/brain/awz371 31782760 PMC6935751

[ref98] Moudy A. M. Kunkel D. D. Malouf A. T. Schwartzkroin P. A. (1993). Development of dopamine-beta-hydroxylase-positive fiber innervation in co-cultured hippocampus-locus coeruleus organotypic slices. Synapse 15, 319–325. 7908762 10.1002/syn.890150408

[ref99] Nelson A. R. Kolasa K. McMahon L. L. (2014). Noradrenergic sympathetic sprouting and cholinergic reinnervation maintains non-amyloidogenic processing of AbetaPP. J Alzheimer's Dis 38, 867–879. doi: 10.3233/JAD-130608 24081376 PMC4047988

[ref100] Nicholson K. J. Sherman M. Divi S. N. Bowles D. R. Vaccaro A. R. (2022). The role of family-wise error rate in determining statistical significance. Clin Spine Surg. 35, 222–223. doi: 10.1097/BSD.0000000000001287, 34907926

[ref101] Oh J. Eser R. A. Ehrenberg A. J. Morales D. Petersen C. Kudlacek J. (2019). Profound degeneration of wake-promoting neurons in Alzheimer's disease. Alzheimers Dement. 15, 1253–1263. doi: 10.1016/j.jalz.2019.06.3916, 31416793 PMC6801040

[ref102] Ohm D. T. Peterson C. Lobrovich R. Cousins K. A. Q. Gibbons G. S. McMillan C. T. . (2020). Degeneration of the locus coeruleus is a common feature of tauopathies and distinct from TDP-43 proteinopathies in the frontotemporal lobar degeneration spectrum. Acta Neuropathol. 140, 675–693. doi: 10.1007/s00401-020-02210-1, 32804255 PMC7554264

[ref103] Park J. Ha H. J. Chung E. S. Baek S. H. Cho Y. Kim H. K. (2021). O-GlcNAcylation ameliorates the pathological manifestations of Alzheimer's disease by inhibiting necroptosis. Sci. Adv. 7:207. doi: 10.1126/sciadv.abd3207, 33523877 PMC7806231

[ref104] Park J. H. Nakamura Y. Li W. Hamanaka G. Arai K. Lo E. H. (2021). Effects of O-GlcNAcylation on functional mitochondrial transfer from astrocytes. J. Cereb. Blood Flow Metab. 41, 1523–1535. doi: 10.1177/0271678X20969588, 33153373 PMC8221762

[ref105] Pekny M. Pekna M. (2014). Astrocyte reactivity and reactive astrogliosis: costs and benefits. Physiol. Rev. 94, 1077–1098. doi: 10.1152/physrev.00041.2013, 25287860

[ref106] Pekny M. Wilhelmsson U. Pekna M. (2014). The dual role of astrocyte activation and reactive gliosis. Neurosci. Lett. 565, 30–38. doi: 10.1016/j.neulet.2013.12.071, 24406153

[ref107] Pentkowski N. S. Berkowitz L. E. Thompson S. M. Drake E. N. Olguin C. R. Clark B. J. (2018). Anxiety-like behavior as an early endophenotype in the TgF344-AD rat model of Alzheimer's disease. Neurobiol. Aging 61, 169–176. doi: 10.1016/j.neurobiolaging.2017.09.024, 29107184 PMC7944488

[ref108] Permanne B. Sand A. Ousson S. Neny M. Hantson J. Schubert R. . (2022). O-GlcNAcase inhibitor ASN90 is a multimodal drug candidate for tau and alpha-Synuclein Proteinopathies. ACS Chem. Neurosci. 13, 1296–1314. doi: 10.1021/acschemneuro.2c00057, 35357812 PMC9026285

[ref109] Phillips S. Chatham J. C. McMahon L. L. (2024). Forskolin reverses the O-GlcNAcylation dependent decrease in GABA(a)R current amplitude at hippocampal synapses possibly at a neurosteroid site on GABA(a)Rs. Sci. Rep. 14:17461. doi: 10.1038/s41598-024-66025-w, 39075105 PMC11286967

[ref110] Powers R. E. Struble R. G. Casanova M. F. O'Connor D. T. Kitt C. A. Price D. L. (1988). Innervation of human hippocampus by noradrenergic systems: normal anatomy and structural abnormalities in aging and in Alzheimer's disease. Neuroscience 25, 401–417. 3399052 10.1016/0306-4522(88)90248-5

[ref111] Preman P. Alfonso-Triguero M. Alberdi E. Verkhratsky A. Arranz A. M. (2021). Astrocytes in Alzheimer's disease: pathological significance and molecular pathways. Cells 10:540. doi: 10.3390/cells10030540, 33806259 PMC7999452

[ref112] Pugh P. L. Vidgeon-Hart M. P. Ashmeade T. Culbert A. A. Seymour Z. Perren M. J. (2007). Repeated administration of the noradrenergic neurotoxin N-(2-chloroethyl)-N-ethyl-2-bromobenzylamine (DSP-4) modulates neuroinflammation and amyloid plaque load in mice bearing amyloid precursor protein and presenilin-1 mutant transgenes. J. Neuroinflammation 4:8. doi: 10.1186/1742-2094-4-8, 17324270 PMC1810243

[ref113] Rodriguez J. J. Olabarria M. Chvatal A. Verkhratsky A. (2009). Astroglia in dementia and Alzheimer's disease. Cell Death Differ. 16, 378–385. doi: 10.1038/cdd.2008.172, 19057621

[ref114] Rodriguez-Gomez J. A. Kavanagh E. Engskog-Vlachos P. Engskog M. K. R. Herrera A. J. Espinosa-Oliva A. M. . (2020). Microglia: agents of the CNS pro-inflammatory response. Cells 9:717. doi: 10.3390/cells9071717, 32709045 PMC7407646

[ref115] Rorabaugh J. M. Chalermpalanupap T. Botz-Zapp C. A. Fu V. M. Lembeck N. A. Cohen R. M. (2017). Chemogenetic locus coeruleus activation restores reversal learning in a rat model of Alzheimer's disease. Brain 140, 3023–3038. doi: 10.1093/brain/awx232, 29053824 PMC5841201

[ref116] Rosas-Arellano A. Villalobos-Gonzalez J. B. Palma-Tirado L. Beltran F. A. Carabez-Trejo A. Missirlis F. . (2016). A simple solution for antibody signal enhancement in immunofluorescence and triple immunogold assays. Histochem. Cell Biol. 146, 421–430. doi: 10.1007/s00418-016-1447-2, 27188756

[ref117] Rostami J. Fotaki G. Sirois J. Mzezewa R. Bergstrom J. Essand M. . (2020). Astrocytes have the capacity to act as antigen-presenting cells in the Parkinson's disease brain. J. Neuroinflammation 17:119. doi: 10.1186/s12974-020-01776-7, 32299492 PMC7164247

[ref118] Sanchez-Mejias E. Navarro V. Jimenez S. Sanchez-Mico M. Sanchez-Varo R. Nunez-Diaz C. . (2016). Soluble phospho-tau from Alzheimer's disease hippocampus drives microglial degeneration. Acta Neuropathol. 132, 897–916. doi: 10.1007/s00401-016-1630-5, 27743026 PMC5106501

[ref119] Scheiderer C. L. McCutchen E. Thacker E. E. Kolasa K. Ward M. K. Parsons D. (2006). Sympathetic sprouting drives hippocampal cholinergic reinnervation that prevents loss of a muscarinic receptor-dependent long-term depression at CA3-CA1 synapses. J. Neurosci. 26, 3745–3756. doi: 10.1523/JNEUROSCI.5507-05.2006, 16597728 PMC6674126

[ref120] Selnick H. G. Hess J. F. Tang C. Liu K. Schachter J. B. Ballard J. E. (2019). Discovery of MK-8719, a potent O-GlcNAcase inhibitor as a potential treatment for Tauopathies. J. Med. Chem. 62, 10062–10097. doi: 10.1021/acs.jmedchem.9b01090, 31487175

[ref121] Shah D. Gsell W. Wahis J. Luckett E. S. Jamoulle T. Vermaercke B. (2022). Astrocyte calcium dysfunction causes early network hyperactivity in Alzheimer's disease. Cell Rep. 40:111280. doi: 10.1016/j.celrep.2022.111280, 36001964 PMC9433881

[ref122] Smith L. A. Goodman A. M. McMahon L. L. (2022). Dentate granule cells are hyperexcitable in the TgF344-AD rat model of Alzheimer's disease. Front. Synaptic Neurosci. 14:826601. doi: 10.3389/fnsyn.2022.826601, 35685246 PMC9171068

[ref123] Smith L. A. McMahon L. L. (2018). Corrigendum to deficits in synaptic function occur at medial perforant path-dentate granule cell synapses prior to Schaffer collateral-CA1 pyramidal cell synapses in the novel TgF344-Alzheimer's disease rat model. Neurobiol. Dis. 118, 177–178. doi: 10.1016/j.nbd.2018.06.011, 29199135 PMC6661255

[ref124] Sofroniew M. V. (2014). Astrogliosis. Cold Spring Harb. Perspect. Biol. 7:a020420. doi: 10.1101/cshperspect.a020420, 25380660 PMC4315924

[ref125] Sofroniew M. V. (2015). Astrocyte barriers to neurotoxic inflammation. Nat. Rev. Neurosci. 16, 249–263. doi: 10.1038/nrn3898, 25891508 PMC5253239

[ref126] Sofroniew M. V. (2020). Astrocyte reactivity: subtypes, states, and functions in CNS innate immunity. Trends Immunol. 41, 758–770. doi: 10.1016/j.it.2020.07.004, 32819810 PMC7484257

[ref127] Stewart L. T. Abiraman K. Chatham J. C. McMahon L. L. (2020). Increased O-GlcNAcylation rapidly decreases GABA(a)R currents in hippocampus but depresses neuronal output. Sci. Rep. 10:7494. doi: 10.1038/s41598-020-63188-0, 32366857 PMC7198489

[ref128] Stewart L. T. Khan A. U. Wang K. Pizarro D. Pati S. Buckingham S. C. (2017). Acute increases in protein O-GlcNAcylation dampen Epileptiform activity in Hippocampus. J. Neurosci. 37, 8207–8215. doi: 10.1523/JNEUROSCI.0173-16.2017, 28760863 PMC5566868

[ref129] Sutter P. A. Crocker S. J. (2022). Glia as antigen-presenting cells in the central nervous system. Curr. Opin. Neurobiol. 77:102646. doi: 10.1016/j.conb.2022.102646, 36371828 PMC10183975

[ref130] Szot P. Leverenz J. B. Peskind E. R. Kiyasu E. Rohde K. Miller M. A. (2000). Tyrosine hydroxylase and norepinephrine transporter mRNA expression in the locus coeruleus in Alzheimer's disease. Brain Res. Mol. Brain Res. 84, 135–140. doi: 10.1016/S0169-328X(00)00168-6, 11113540

[ref131] Szot P. White S. S. Greenup J. L. Leverenz J. B. Peskind E. R. Raskind M. A. (2006). Compensatory changes in the noradrenergic nervous system in the locus ceruleus and hippocampus of postmortem subjects with Alzheimer's disease and dementia with Lewy bodies. J. Neurosci. 26, 467–478. doi: 10.1523/JNEUROSCI.4265-05.2006, 16407544 PMC6674412

[ref132] Szot P. White S. S. Greenup J. L. Leverenz J. B. Peskind E. R. Raskind M. A. (2007). Changes in adrenoreceptors in the prefrontal cortex of subjects with dementia: evidence of compensatory changes. Neuroscience 146, 471–480. doi: 10.1016/j.neuroscience.2007.01.031, 17324522 PMC3399726

[ref133] Taddei R. N. Perbet R. Mate de Gerando A. Wiedmer A. E. Sanchez-Mico M. Connors Stewart T. (2023). Tau oligomer-containing synapse elimination by microglia and astrocytes in Alzheimer disease. JAMA Neurol. 80, 1209–1221. doi: 10.1001/jamaneurol.2023.3530, 37812432 PMC10562992

[ref134] Taddei R. N. Sanchez-Mico M. V. Bonnar O. Connors T. Gaona A. Denbow D. (2022). Changes in glial cell phenotypes precede overt neurofibrillary tangle formation, correlate with markers of cortical cell damage, and predict cognitive status of individuals at Braak III-IV stages. Acta Neuropathol. Commun. 10:72. doi: 10.1186/s40478-022-01370-3, 35534858 PMC9082857

[ref135] Tan C. X. Eroglu C. (2021). Cell adhesion molecules regulating astrocyte-neuron interactions. Curr. Opin. Neurobiol. 69, 170–177. doi: 10.1016/j.conb.2021.03.015, 33957433 PMC8387342

[ref136] Taylor E. W. Wang K. Nelson A. R. Bredemann T. M. Fraser K. B. Clinton S. M. (2014). O-GlcNAcylation of AMPA receptor GluA2 is associated with a novel form of long-term depression at hippocampal synapses. J. Neurosci. 34, 10–21. doi: 10.1523/JNEUROSCI.4761-12.2014, 24381264 PMC3866478

[ref137] Theofilas P. Ehrenberg A. J. Nguy A. Thackrey J. M. Dunlop S. Mejia M. B. (2018). Probing the correlation of neuronal loss, neurofibrillary tangles, and cell death markers across the Alzheimer's disease Braak stages: a quantitative study in humans. Neurobiol. Aging 61, 1–12. doi: 10.1016/j.neurobiolaging.2017.09.007, 29031088 PMC5705284

[ref138] Town T. Nikolic V. Tan J. (2005). The microglial "activation" continuum: from innate to adaptive responses. J. Neuroinflammation 2:24. doi: 10.1186/1742-2094-2-24, 16259628 PMC1298325

[ref139] Umapathi P. Mesubi O. O. Banerjee P. S. Abrol N. Wang Q. Luczak E. D. (2021). Excessive O-GlcNAcylation causes heart failure and sudden death. Circulation 143, 1687–1703. doi: 10.1161/CIRCULATIONAHA.120.051911, 33593071 PMC8085112

[ref140] Umpierre A. D. Wu L. J. (2021). How microglia sense and regulate neuronal activity. Glia 69, 1637–1653. doi: 10.1002/glia.23961, 33369790 PMC8113084

[ref141] Wahis J. Holt M. G. (2021). Astrocytes, noradrenaline, alpha1-Adrenoreceptors, and Neuromodulation: evidence and unanswered questions. Front. Cell. Neurosci. 15:645691. doi: 10.3389/fncel.2021.645691 33716677 PMC7947346

[ref142] Wang X. Li W. Marcus J. Pearson M. Song L. Smith K. (2020). MK-8719, a novel and selective O-GlcNAcase inhibitor that reduces the formation of pathological tau and ameliorates neurodegeneration in a mouse model of Tauopathy. J. Pharmacol. Exp. Ther. 374, 252–263. doi: 10.1124/jpet.120.266122, 32493725

[ref143] Wang D. Yuen E. Y. Zhou Y. Yan Z. Xiang Y. K. (2011). Amyloid beta peptide-(1-42) induces internalization and degradation of beta2 adrenergic receptors in prefrontal cortical neurons. J. Biol. Chem. 286, 31852–31863. doi: 10.1074/jbc.M111.244335, 21757762 PMC3173113

[ref144] Wang M. Zhu W. Guo Y. Zeng H. Liu J. Liu J. . (2024). *Astragalus* polysaccharide treatment relieves cerebral ischemia–reperfusion injury by promoting M2 polarization of microglia by enhancing O-GlcNAcylation. Metab. Brain Dis. 40:16. doi: 10.1007/s11011-024-01420-w, 39560836

[ref145] Webers A. Heneka M. T. Gleeson P. A. (2020). The role of innate immune responses and neuroinflammation in amyloid accumulation and progression of Alzheimer's disease. Immunol. Cell Biol. 98, 28–41. doi: 10.1111/imcb.12301, 31654430

[ref146] Wegiel J. Wang K. C. Tarnawski M. Lach B. (2000). Microglia cells are the driving force in fibrillar plaque formation, whereas astrocytes are a leading factor in plague degradation. Acta Neuropathol. 100, 356–364. doi: 10.1007/s004010000199, 10985692

[ref147] Weinshenker D. (2018). Long road to ruin: noradrenergic dysfunction in neurodegenerative disease. Trends Neurosci. 41, 211–223. doi: 10.1016/j.tins.2018.01.010, 29475564 PMC5878728

[ref148] Weinshenker D. Ferrucci M. Busceti C. L. Biagioni F. Lazzeri G. Liles L. C. (2008). Genetic or pharmacological blockade of noradrenaline synthesis enhances the neurochemical, behavioral, and neurotoxic effects of methamphetamine. J. Neurochem. 105, 471–483. doi: 10.1111/j.1471-4159.2007.05145.x, 18042179 PMC2610530

[ref149] Wendimu M. Y. Hooks S. B. (2022). Microglia phenotypes in aging and neurodegenerative diseases. Cells 11:2091. doi: 10.3390/cells11132091, 35805174 PMC9266143

[ref150] Wheatley EG Albarran E White CW, 3rd, Bieri G Sanchez-Diaz C Pratt K . (2019). Neuronal O-GlcNAcylation improves cognitive function in the aged mouse brain. Curr. Biol. 29, 3359–69 e4. doi: 10.1016/j.cub.2019.08.003 31588002 PMC7199460

[ref151] Wu Y. G. Song L. J. Yin L. J. Yin J. J. Wang Q. Yu J. Z. (2023). The effects and potential of microglial polarization and crosstalk with other cells of the central nervous system in the treatment of Alzheimer's disease. Neural Regen. Res. 18, 947–954. doi: 10.4103/1673-5374.355747, 36254973 PMC9827789

[ref152] Xie L. Zhang N. Zhang Q. Li C. Sandhu A. F. Iii G. W. . (2020). Inflammatory factors and amyloid beta-induced microglial polarization promote inflammatory crosstalk with astrocytes. Aging (Albany NY) 12, 22538–22549. doi: 10.18632/aging.103663 33196457 PMC7746366

[ref153] Yao H. Liang C. Wang X. Duan C. Song X. Shang Y. (2025). OGT-mediated O-GlcNAcylation of ATF2 protects against Sepsis-associated encephalopathy by inhibiting microglial Pyroptosis. Neurosci. Bull. 41, 1761–1778. doi: 10.1007/s12264-025-01418-z, 40411666 PMC12494526

[ref154] Yuzwa S. A. Shan X. Jones B. A. Zhao G. Woodward M. L. Li X. (2014). Pharmacological inhibition of O-GlcNAcase (OGA) prevents cognitive decline and amyloid plaque formation in bigenic tau/APP mutant mice. Mol. Neurodegener. 9:42. doi: 10.1186/1750-1326-9-42, 25344697 PMC4232697

[ref155] Zhao P. Viner R. Teo C. F. Boons G. J. Horn D. Wells L. (2011). Combining high-energy C-trap dissociation and electron transfer dissociation for protein O-GlcNAc modification site assignment. J. Proteome Res. 10, 4088–4104. doi: 10.1021/pr2002726, 21740066 PMC3172619

[ref156] Zheng G. M. Yu C. Yang Z. (2012). Puerarin suppresses production of nitric oxide and inducible nitric oxide synthase in lipopolysaccharide-induced N9 microglial cells through regulating MAPK phosphorylation, O-GlcNAcylation and NF-kappaB translocation. Int. J. Oncol. 40, 1610–1618. doi: 10.3892/ijo.2012.1331 22246431

[ref157] Zhou B. Zuo Y. X. Jiang R. T. (2019). Astrocyte morphology: diversity, plasticity, and role in neurological diseases. CNS Neurosci. Ther. 25, 665–673. doi: 10.1111/cns.13123, 30929313 PMC6515705

[ref158] Zong C. Sato H. Schneider B. Shichino S. Ueha S. Wu B. . (2025). Acrylamide-induced noradrenergic axon degeneration is promoted via a non-cell autonomous mechanism, involving microglial Tnfaip2/TNF-alpha and oxidative stress pathways. J. Hazard. Mater. 496:139125. doi: 10.1016/j.jhazmat.2025.139125 40664071

