# Supplementary figures and images for: Pharmacologically increasing O-GlcNAcylation increases complexity of astrocytes in the dentate gyrus of TgF344-AD rats
Source: Front Aging Neurosci. 2025 Dec 16;17:1690410. doi: 10.3389/fnagi.2025.1690410 (PMC12748250; doi:10.3389/fnagi.2025.1690410)

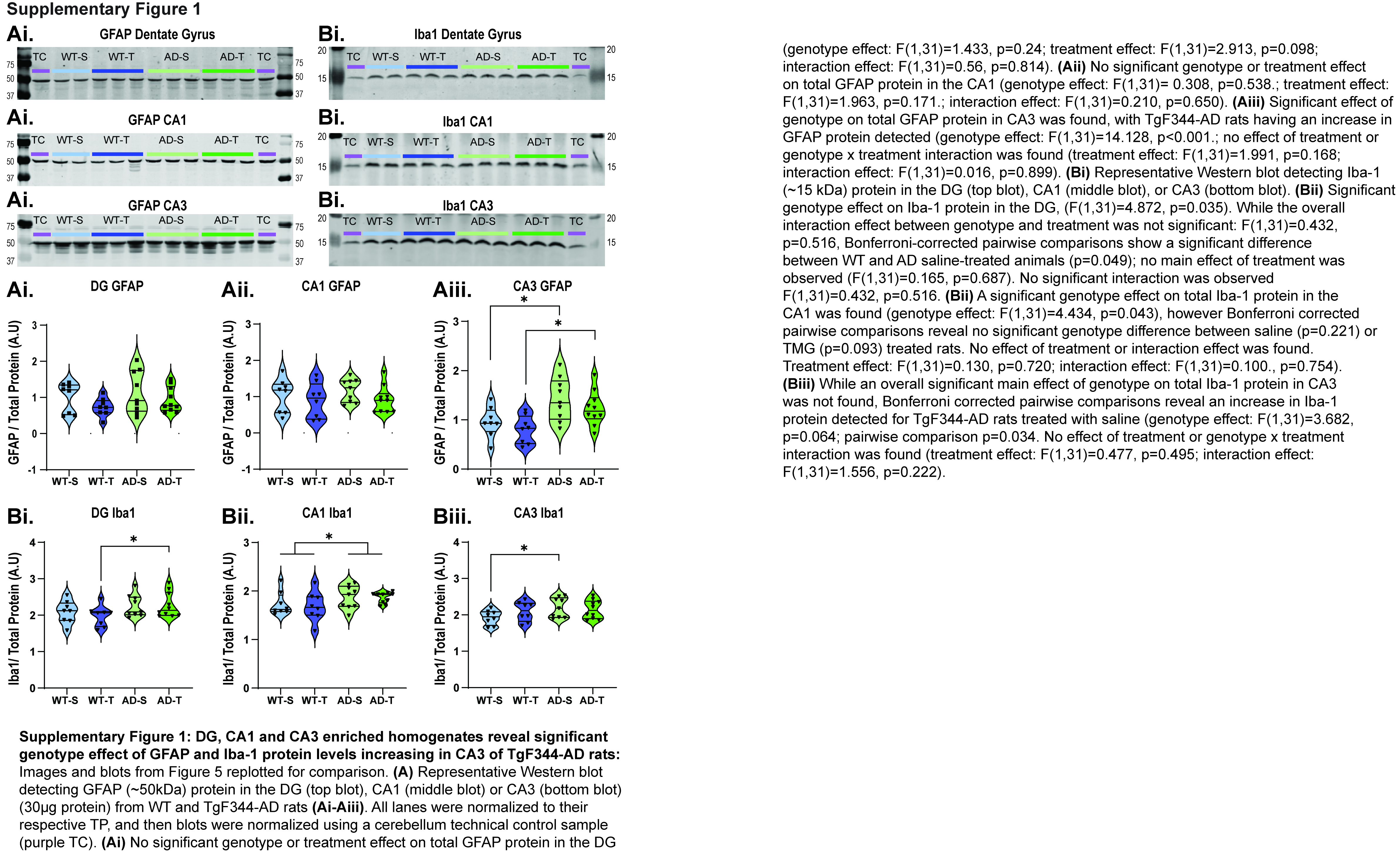

Supplement: Supplementary file 1 [file Image_1.tif]

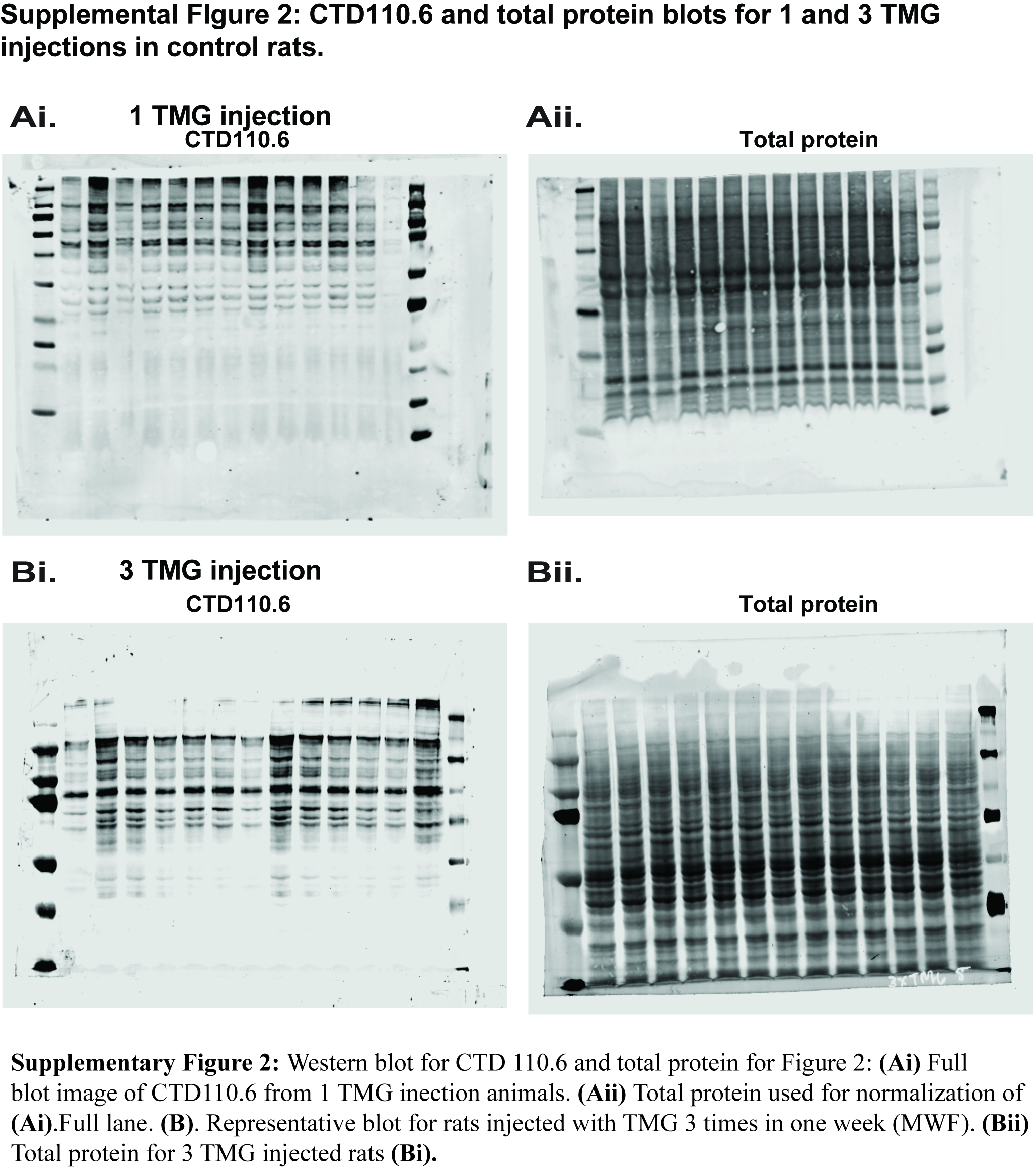

Supplement: Supplementary file 2 [file Image_2.tif]

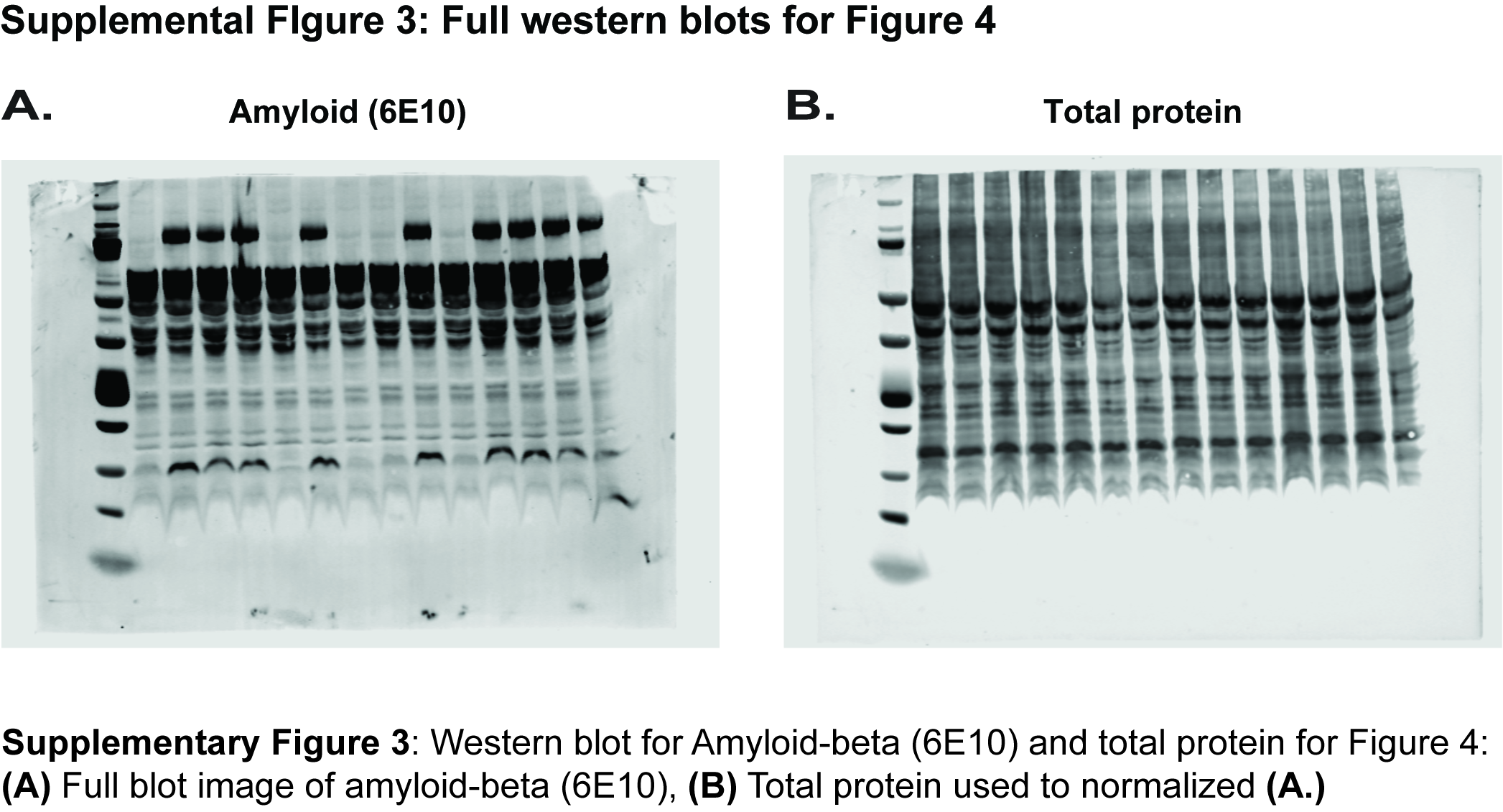

Supplement: Supplementary file 3 [file Image_3.tif]

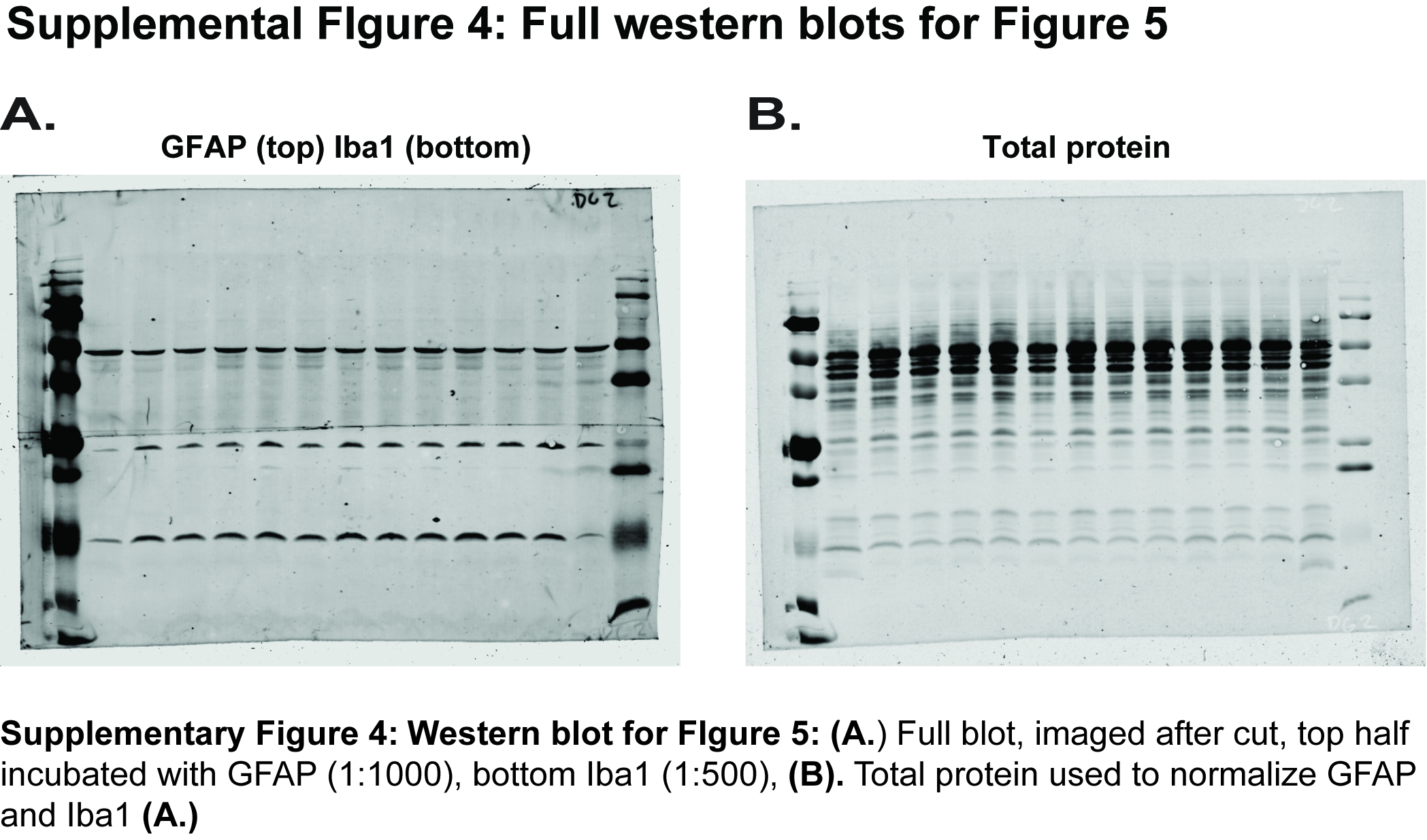

Supplement: Supplementary file 4 [file Image_4.tif]

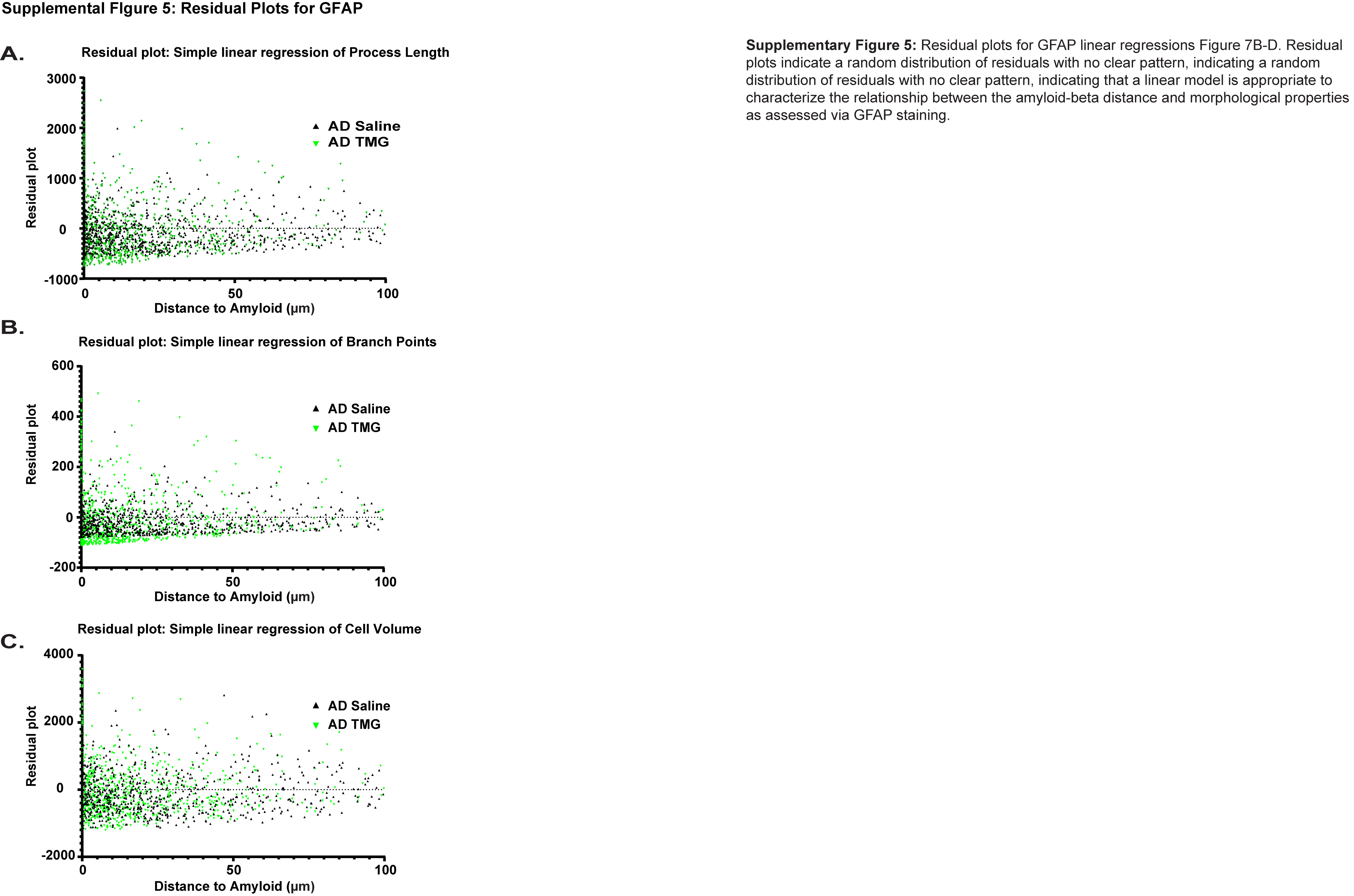

Supplement: Supplementary file 5 [file Image_5.tif]

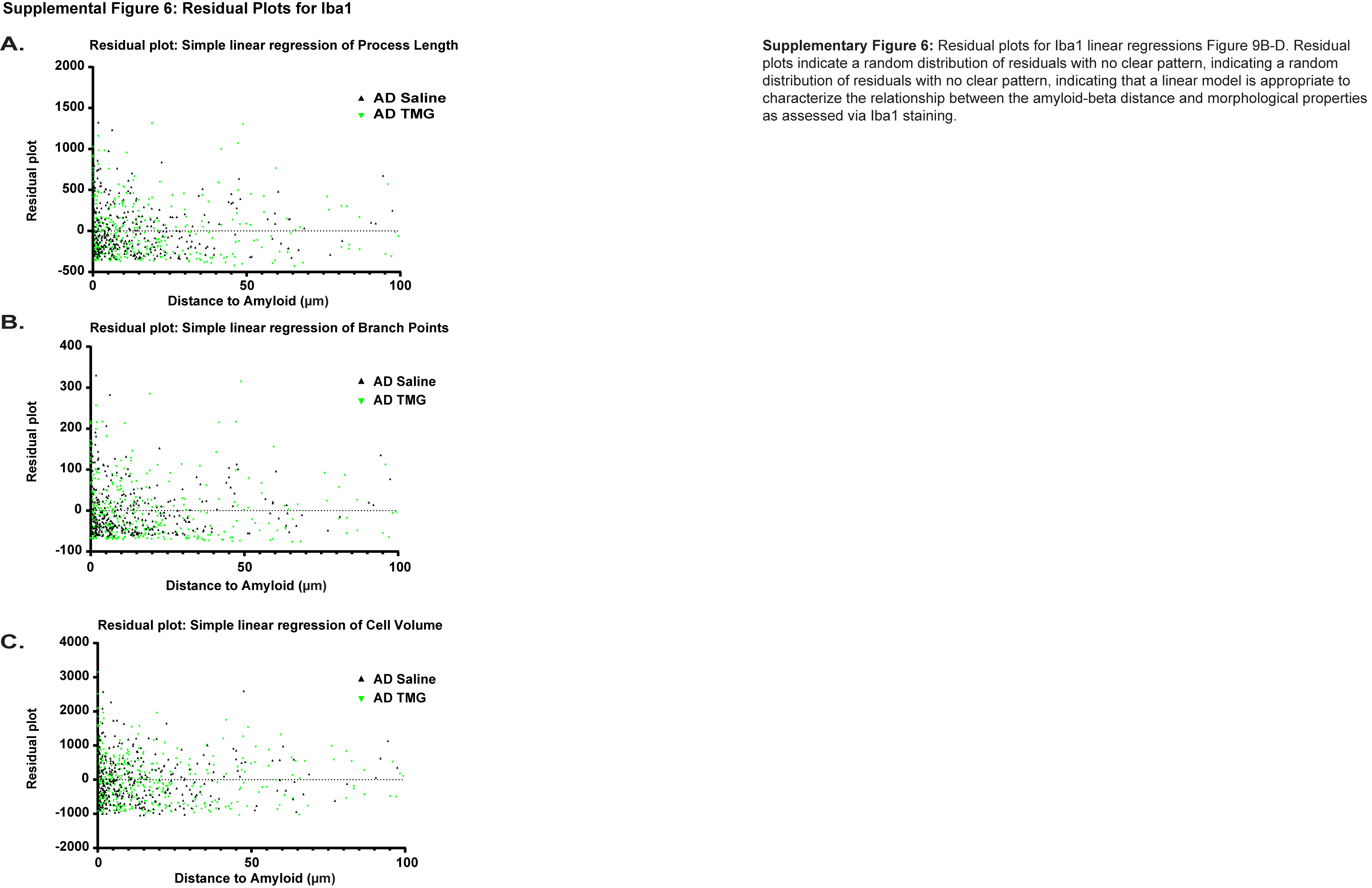

Supplement: Supplementary file 6 [file Image_6.tif]
